# Physico-analytical studies on some heterocyclic azo dyes and their metal complexes with some transition metals

**DOI:** 10.1186/s13065-022-00833-x

**Published:** 2022-05-30

**Authors:** Fathy A. El-Seify, Hassan A. Azab, Fikrea S. Degedy, Khalid A. Abdel-Mageed, Farid I. El-Dossoki

**Affiliations:** 1 Chemistry Department, Faculty of Engineering, Port Said University, Port Said, Egypt; 2 Chemistry Department, Faculty of Science, Suez Canal University, Ismalia, Egypt; 3 Chemistry Department, Faculty of Science, Port Said University, Port Said, Egypt

**Keywords:** Heteroaromatic amines, Azo dye, Spectrophotometric, Chromogenic reagents

## Abstract

In this investigation, the azo dyes; 2-(3′-phenyl-5′-pyrazolyl azo) schaffer acid (la) and 2-(3′-phenyl-5′-pyrazolyl azo) resorcinol (Ib); were prepared through diazotizing 3-phenyl-5-aminopyrazole (PAP) and coupling the resulting diazonium salt with Schäffer acid and resorcinol respectively. The prepared azo dyes are characterized using both IR spectra and the elemental analysis (C, H, N and S). The prepared azo dyes are used as chromogenic reagents for the spectrophotometric determination of copper (II), nickel (II), cobalt (II) and zinc (II) ions. The conditional acid dissociation constants of these azo dyes (la and Ib) and the stability constants of its metal ion complexes have been determined by spectro-analytical methods. The effect of pH, time, organic solvent and the foreign ions on the spectrophotometric determination of these ions and their complexes with the azo dyes under study were studied. The stoichiometric ratio (M:L) of the formed complexes was also determined. The molar absorptivity, the Sandell's sensitivity values, the obeyance of Beers law and the stability constants of the formed complexes have been also determined and discussed.

## Introduction

The chemistry of heteroaromatic compounds is a very important part of the wide field of organic chemistry. Perhaps one of the most unusual facts of the chemistry of heterocyclic compounds is the enormous literature reported for pyrazole derivatives. The interesting dyeing properties of many azopyrazole derivatives as well as the early discovery of the very influential biological activity, especially antibacterial and anticancer properties of aminopyrazoles and anthrapyrazoles have definitely contributed to this unusual interest [1–4].

All azo compounds contain at least one, but more usually two, aromatic residues attached to the azo group. They exist in the more stable trans-form rather than the cis-form. The two nitrogen atoms are assumed to be sp^2^ hybridized and the remaining p-electrons are used to build up the $\pi$ -orbital between them. This leads to coplanar but non-linear arrangement for azo compound. The parent substance for this class of compounds in diimide HN = NH, a fungitive material about which relatively little is known, compared to the isoelectronic systems ethylene and formaldehyde. Azo compounds are more stable than hydrazo compounds and therefore a good number of different structures of azo compounds have been determined. Most azo molecules are planar with a reference for the transform [3–5].

Azo dyes which form the largest group of organic dyes, constitute more than 35% of the world production of all dyes and thus are becoming extensively scattered throughout the environment around manufacturing plants. For many years ago metal chelates of azo dyes have been used as dyes and indicators as chromophoric reagents in the spectrophotometric determination of metal ions; copper (II), nickel (II), cobalt (II) and zinc (II) [1–5]. 3-phenyl-5-aminopyrazole with its very stable diazonium salt was found to be a very active organic heteroamines as a start for many organic preparations, but until now, there is no applications that have been detected at analytical chemistry fields.

To date mainly spectrophotometric and chromatographic methods have been used for the analysis of the azo dyes and for their use in the determination of the transition metal ions [1–15]. Therefore, the appropriate sensitivity of the spectrophotometric methods, their applicability over an unusual wide concentration range, and their low coast, are highly satisfactory for meeting requirements of toxicological, ecotoxicological and the environmental regulation enforcement. The present work deals with reporting a new chromogenic reagents and new acid–base indicators {2-(3′-phenyl-5′-pyrazolyl azo) schaffer acid (la) and 2-(3′-phenyl-5′-pyrazolyl azo) resorcinol (Ib)} for the spectrophotometric determination of copper (II), nickel (II), cobalt (II) and zinc (II) ions.

## Experimental

### Materials and solutions

#### Materials

All chemicals used in this investigation were as presented in Table 1. Bidistilled water was used.

**Table 1:** Chemical sample data for the chemical compounds used

Compound	Reg. CAS Number	suppliers	%Purity before purification	Purification method	%Purity before purification
Co(NO_3_)_2_	7791-13-1	Merck Darmstadt	(99.0%)	The compounds were used without further purification	(99.0%)
Ni (NO_3_)_2_	7718-54-9	Merck Darmstadt	(98.0%)		(98.0%)
Cu (NO_3_)_2_	744-39-4	Merck Darmstadt	(97.8%)		(97.8%)
Zn(NO_3_)_2_	7646-85-7	Merck Darmstadt	(97.0%)		(97.0%)
Methanol	64-17-6	Sigma Aldrich	(97.8%)		(97.8%)
Ethanol	64-17-5		(97.8%)		(97.8%)
Isopropanol	178-18-7		(97.9%)		(97.9%)
DMSO	232-11-1		(≥ 97.8%)		(≥ 97.8%)
DMF	211-12-7		(98.7%)		(98.7%)
CHCl_3_	89-15-3		(98.2%)		(98.2%)
Acetone	77-22-6		(97.9%)		(97.9%)

#### Solutions

Stock (0.001 mol dm^−3^) solutions of the Ia and Ib azo dyes under test were prepared by dissolving the accurately weighed amount in the appropriate volume of absolute ethyl alcohol. Solutions of lower concentrations were prepared daily by diluting the stock solution with highly purified ethanol. Stock solutions (0.01 mol dm^−3^) of the transition metals nitrates were prepared using bidistilled water and the concentrations were determined by titration with standardized ethylene diamine tetra acetic acid disodium salt (EDTA) [16]; Lower concentrations were prepared by accurate dilution to the needed volume in a standard flask with bidistilled water.

A series of universal buffer solutions covering the pH-range 2.0 to 13.0 was prepared as recommended by Britton. [17] 0.01 mol dm^−3^ EDTA was prepared by dissolving the calculated amount of AR disodium salt of EDTA in bidistilled water. The solutions of the interfering cations and anions (1 mg/ml) were prepared in bidistilled water. Organic solvents used in this investigation (methyl, ethyl and isopropyl alcohols, acetone, chloroform and DMF) were purified according to recommended methods [18].

### Apparatus

The spectrophotometric measurements were carried out using UV/Visible/near IR double beam spectrophotometer model Jusco V-570, with Quartz 1-cm cells. The pH measurements were made on solutions in a double-walled glass vessel at (25 $\pm$ 0.1 °C) with a commercial Fisher combination electrode (catalog no.13–639-104) containing calomel reference electrode. A Fisher Accumet pH/ion meter model 825 MP was used. The conductometric measurements were carried out using a conductivity bridge of a type (JENWAY).

### Synthesis of 3-phenyl-5-aminopyrazole azo dyes Ia and Ib:

To a stirred cold solution of 3-phenyl-5-aminopyrazolediazonium chloride (0.005 mol) in ethanol (40 ml) add an equivalent amount (0.005 mol) of Schäffer acid (la) or resorcinol (Ib)] portion wise over a period of 30 min. After stirring the reaction mixture for further 2 h, the precipitated solid was filtered and washed with water. Crystallization from ethanol give pure 2-(3′-phenyl-5′-pyrazolyl azo) schaffer acid (la) (MW = 394.346 g/mole), or 2-(3′-phenyl-5′-pyrazolyl azo) resorcinol (Ib) (MW = 280.22 g/mole), which are dried in a vacuum desiccator over anhydrous CaCl_2_. The two azo dyes (Ia and Ib) have been investigated by elemental analysis (C, H, N and S elements) and IR spectra. The analysis of (C, H, N, and S) and IR spectra are determined on the micro-analytical unit at the Chemistry Department, Faculty of science, Cairo University.

**Fig. 1:**
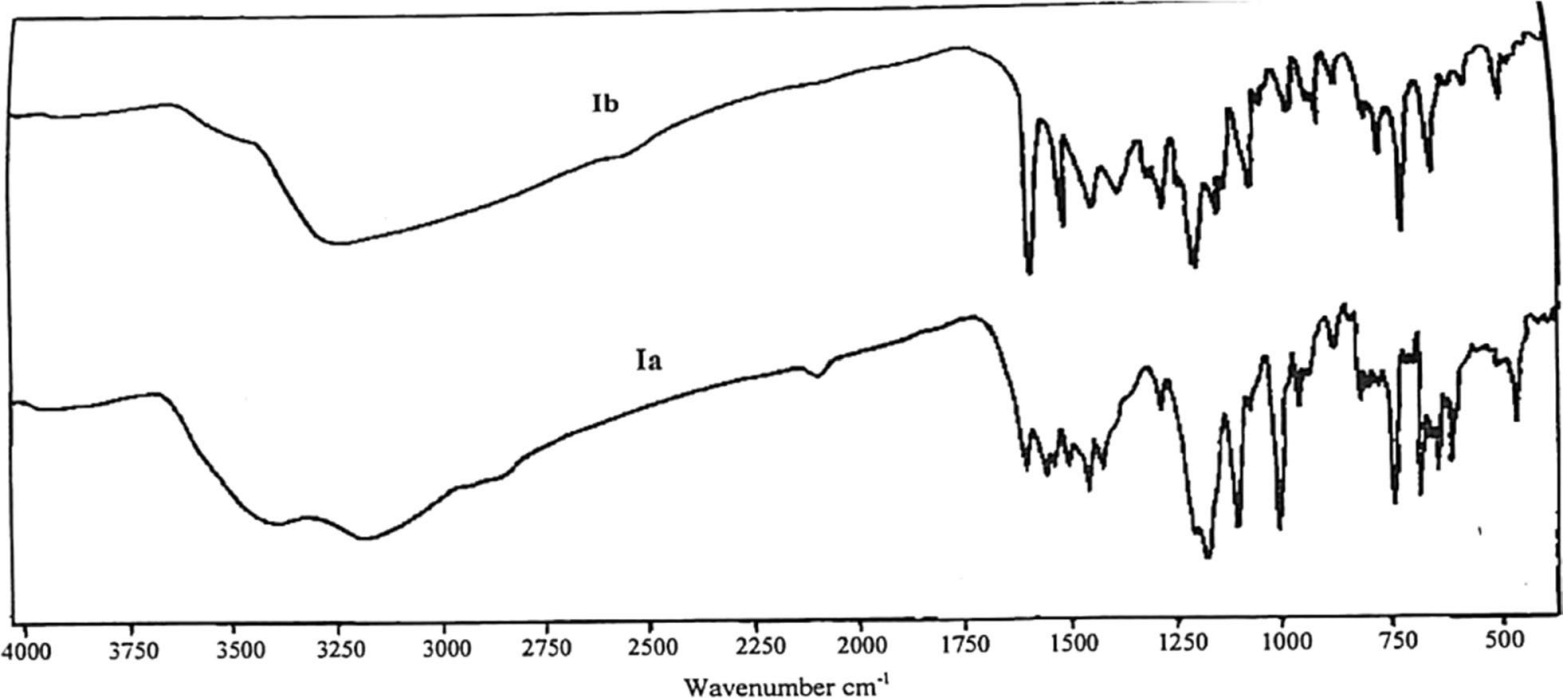
IR spectra of the azo dye ligands Ia and Ib

The two dyes (la and Ib) are sparingly soluble in water while they are soluble in ethanol, methanol, acetone, and chloroform and easily soluble in DMF, dimethyl sulfoxide (DMSO). Ia azo dye found to be red in alkaline solution, orange in weakly acidic solution, and yellow in strongly acidic solution, while the Ib color was found as purple-red in strongly alkaline solution, yellowish-orange in weakly acidic solution, and clear yellow in strong acidic solution. These test which is made using HCl and ammonia gives an implication that they can be easily used as acid–base indicators. The two synthesized PAP azo dyes (la and Ib) are well characterized by elemental analysis as depicted in Table 2 and IR spectra (Fig. 1).

**Table 2:** Experimental data of the **e**lemental analysis of PAP azo dyes (Ia and Ib)

*Azo dye*	Molecular weight	Colour	M.P	Elemental analysis Calculated Found							
				C%	H%	N%	S%	C%	H%	N%	S%
Ia	394.346	Bright red	$>$ 350 $^\circ{\rm C}$	57.82	3.58	14.21	8.13	57.67	3.61	14.17	8.15
Ib	280.22	Orange	198 $^\circ{\rm C}$	64.29	4.32	19.99	–	64.24	4.37	20.4	–

Standard uncertainties, u, are: u(C %) = 0.02, u(H %) = 0.03, u(N %) = 0.02, u(S %) = 0.01

The IR spectra of PAP azo-dyes (Ia and Ib) show the presence of; two broad bands at 3408 (in Ia) and 3270 (in Ib) assignment for $v$ OH group and two strong bands at 3066 (in Ia) and 3149 (in Ib) assignment for $v$ NH group. Two strong bands at 1567 (in Ia) and 1622 (in Ib) assignment for overlapping with C = N. Two strong bands at 1473 (in Ia) and 1476 (in Ib) assignment for $v$ N = N. Two strong bands at 1186 (in Ia) and 1233 (in Ib) assignment for $\delta$ OH group. Two strong bands at 1120 (in Ia) and 1110 (in Ib) assignment for $\gamma$ CH group. One strong band at 1037 (in Ia) assignment for $v$ SO_3_H group.

### Methods applied for determination of the molecular structure of metal-azo dye complexes

The molecular structure of metal-azo dye complexes were determined applying both the molar ratio method [19] and continuous variation method [20, 21].

## Results and discussion

### Spectrophotometric determination of PAP azo dyes (la and Ib) and its transition metal chelates

From the above characterization of (Ia and Ib) azo dyes, the structures of the two concerned ligands are as follow:
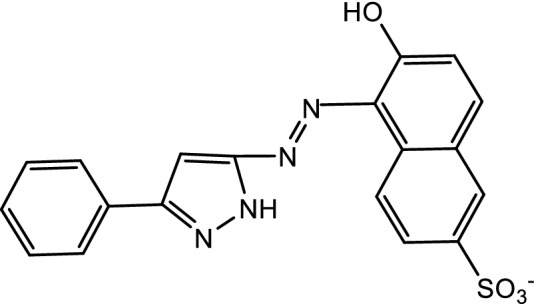


[2-(3′-phenyl-5′-pyrazolyl azo) Schaffer acid (la)].
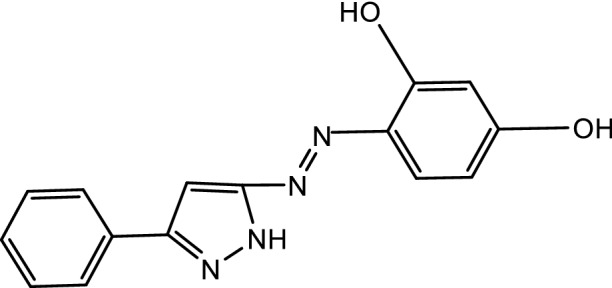


[2-(3′-phenyl-5′ -pyrazolyl azo) resorcinol (Ib)].

#### Effect of pH on the absorption spectra of the azo compounds Ia, Ib

**Fig. 2:**
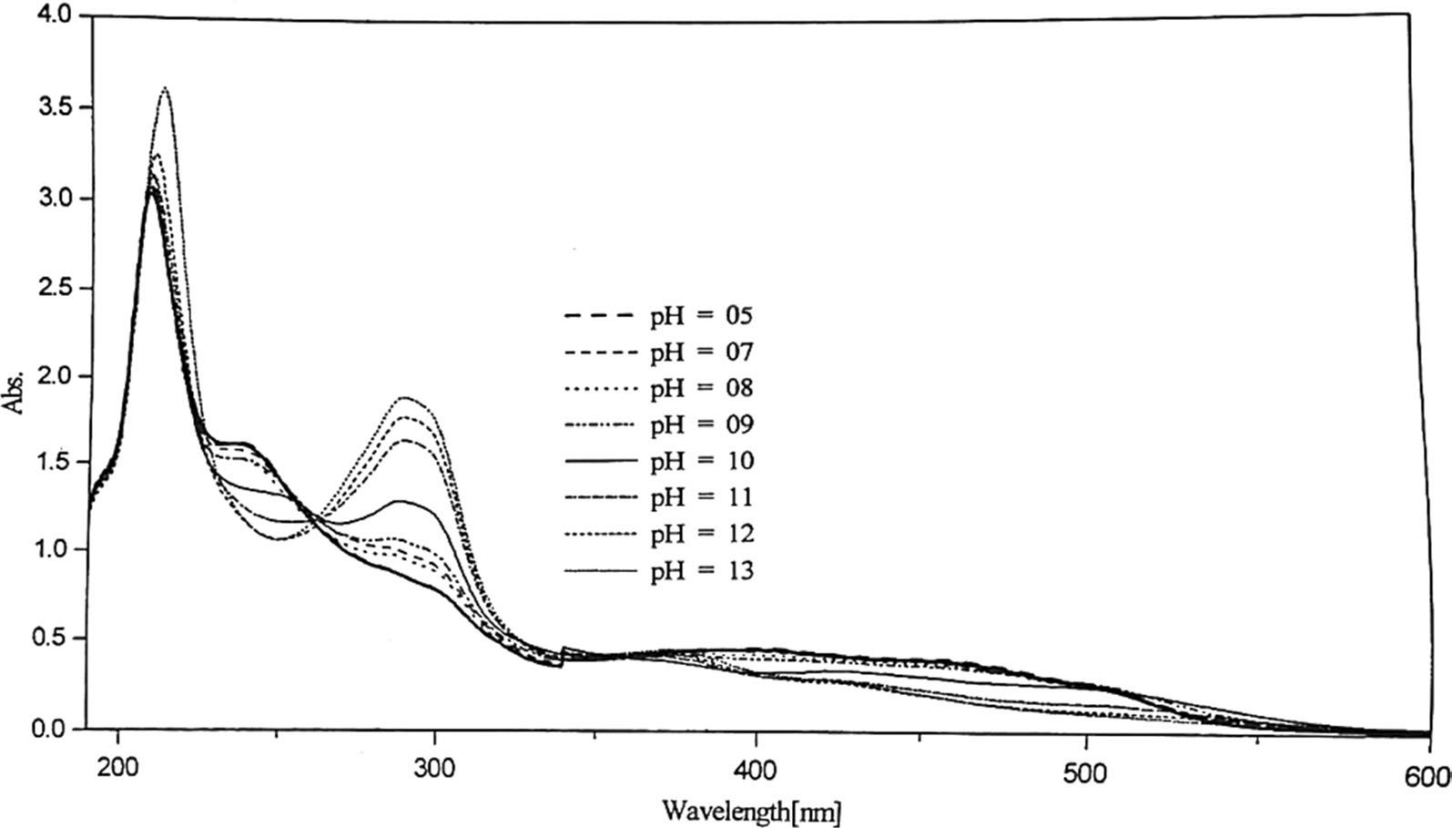
Effect of pH on the absorption spectra of 5 × 10^–5^ mol dm^−3^ of Ia azo dye

This study is used to throw light on the species that may be formed in solutions of different pH's. Thus it can help us to calculate the dissociation constants of such compounds. The absorption spectra of 1 × 10^–4^ mol dm^−3^ of Ia and Ib azo dyes were scanned within the wavelength range 190–600 nm at different pH values. For Ia compound, Fig. 2, three characteristic bands are observed in the vicinity of 213, 289 and 460 nm. Two isosbestic points are detected at 262 and 354 nm at nearly all the pH’s used.

**Fig. 3:**
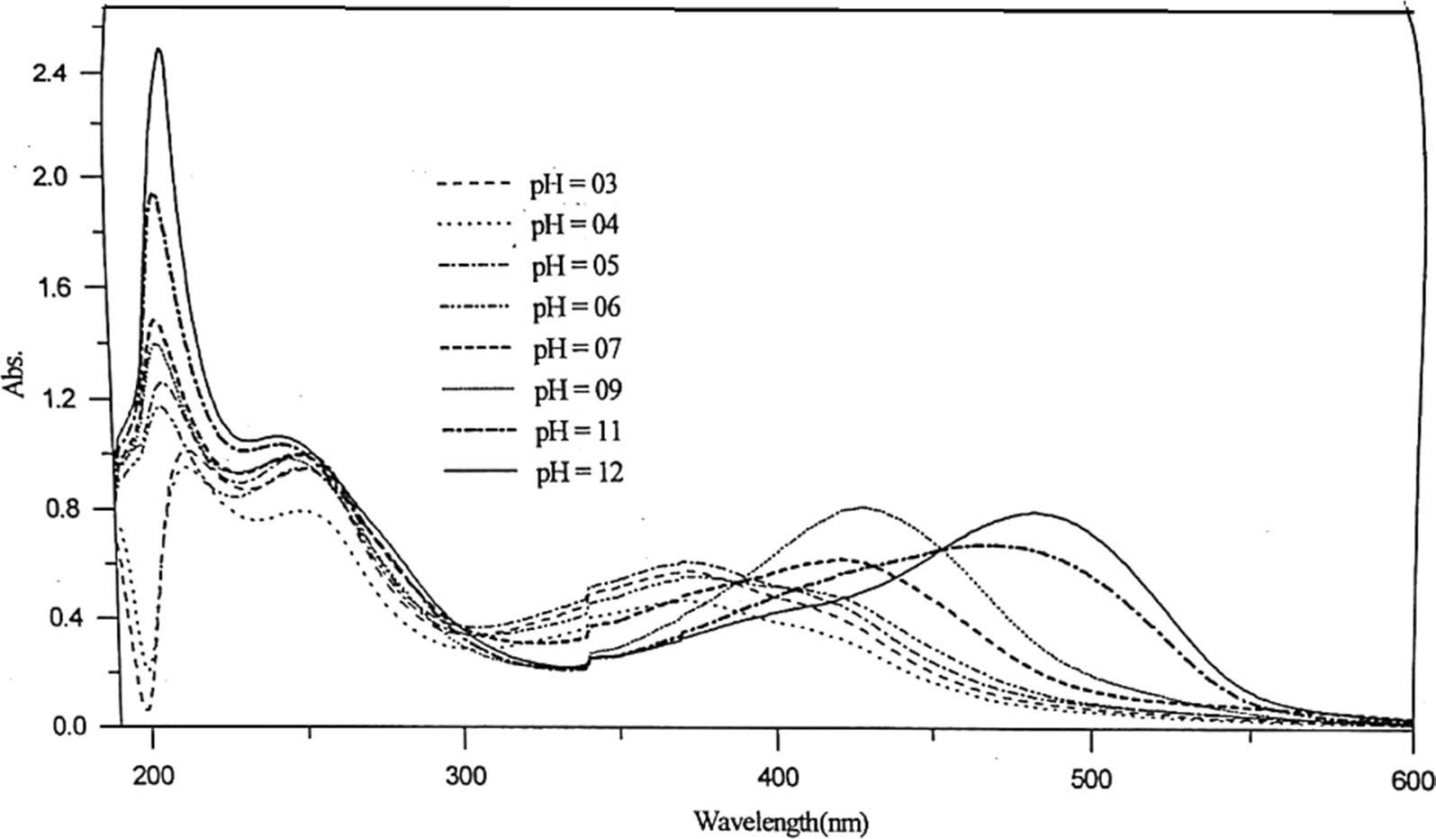
Effect of pH on the absorption spectra of 5 × 10^–5^ mol dm^−3^ of Ib azo dye

In the case of Ib (Fig. 3), slight red shift as the pH increases compared to that of the former compound (Ia) with the existence of two bands; the first at 210 nm and the other at the vicinity of the region 370–465 nm. An isosbestic point at 303 nm was noted indicate the existence of only two, absorbing species, usually in equilibrium with each other [22]. The isosbestic point may occur in a system containing several absorbing species where the variation in concentration of all these species is related by a single reaction parameter i.e. this point can be performed in systems in which simultaneous reaction gives two or more absorbing products, but they cannot be performed in systems in which a reaction of the type A → B → C takes place [22]. The band near 210 nm is due to $\pi - {\pi }^{*}$ transition of the phenyl ring of this compound, it increases in intensity as the pH increases with high $\varepsilon$ values.

Due to the week basicity of the azo group, protonation of the azo group may take place in strong acidic media with the formation of a protonated species. The stepwise acid–base [23] ionization of the Ib compound, referring to Fig. 3, indicate that the ionization of the two hydroxyl groups and pyrazole nitrogen proceeds in different steps as the pH increase. The yellow color of the dye solution changes to dark orange at pH 7, assuming that the ionization of the free H_1_ occurs firstly in pH range 4–6. After that, ionization of the ortho group; H_2_ takes place, leading to the disturbance of the chromophoric azo group arrangement in the intramolecular hydrogen bonding structure.

**Scheme 1:**
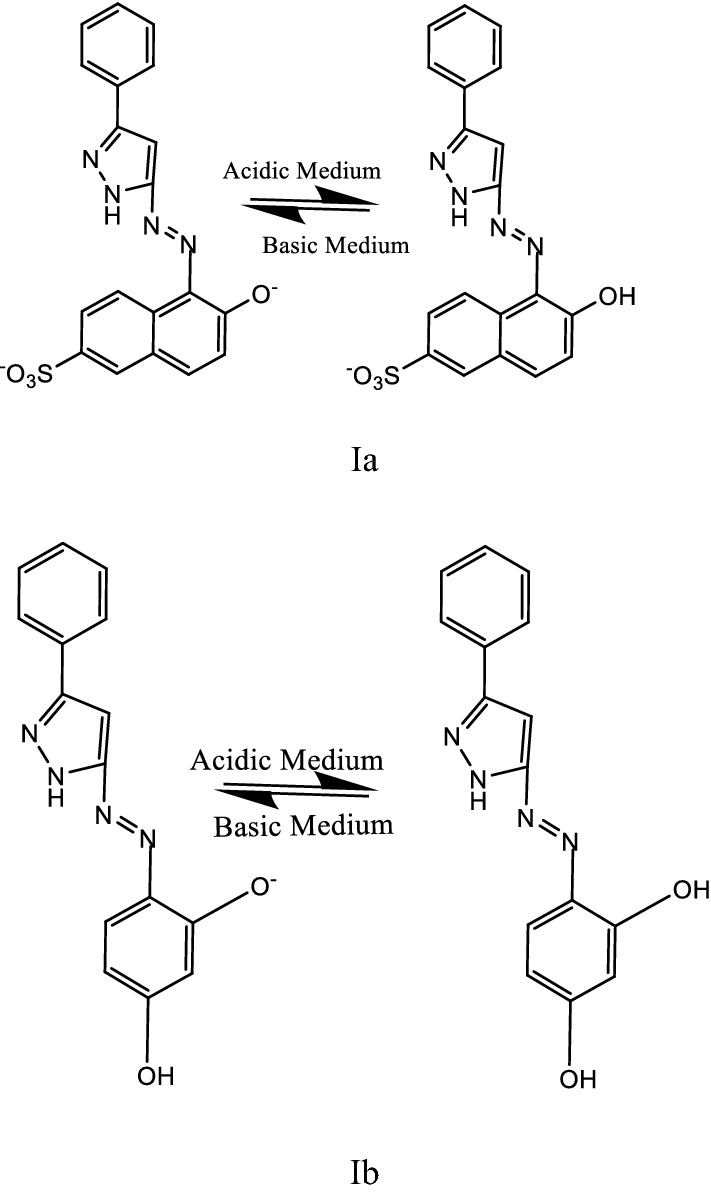
The equilibrium of the dissociation for ligands Ia and Ib

The equilibrium of the dissociation for ligands Ia and Ib can be shown as in Scheme 1.

**Fig. 4:**
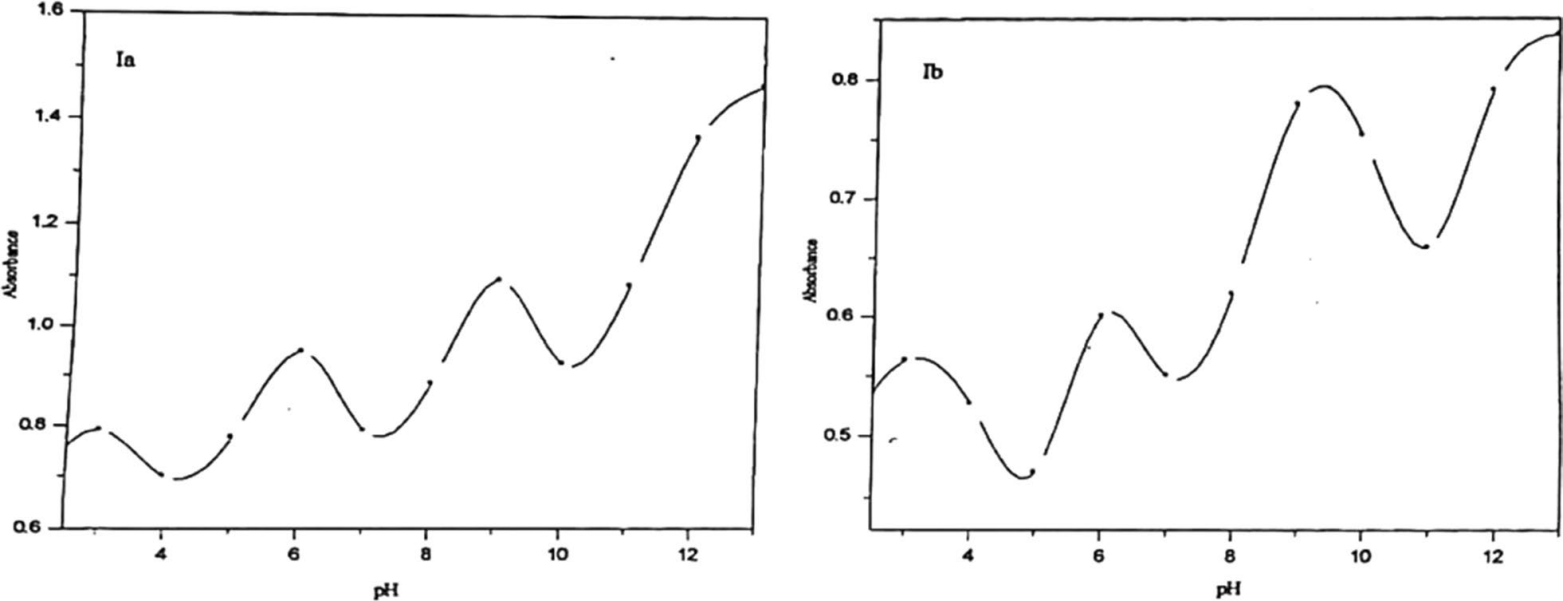
Absorption-pH curves of 5 × 10^–5^ mol dm^−3^ of Ia and Ib azo dyes

The absorbance-pH curves at the selected wavelengths were presented in Fig. 4. The first step within the pH range (2.0–6.5) may represent the deprotonation of the pyrazolic nitrogen (N2) and the ionization of the Trans group (SO_3_H for Ia, OH for Ib). The second step, within the pH range (6.5–9.5), represents the ionization of the cis OH group. The third step within the pH range (10.0–13.0), represents the ionization of the NH group of pyrazole moiety.

#### Determination of the dissociation constant of la and Ib azo-compounds from the spectrophotometric measurements

The change of absorbance with pH can be utilized for the determination of the dissociation constants of the compounds under investigation. The dissociation constants of the ionizable groups of the aminopyrazole azo dyes (la and Ib) were evaluated by applying the recommended spectrophotometric methods; Half height and limiting absorbance methods [24]. The two methods are efficient for the calculation of pK_1_, pK_2_, and pK_3_ (Table 3).

**Table 3:** Experimental data of the dissociation constants of Ia and Ib using half height method (H.H.M) and limiting absorbing method (L.A.M)

**Dye**	pK_1_		pK_2_		pK_3_	
	H.H.M	L.A.M	H.H.M	L.A.M	H.H.M	L.A.M
Ia	5.21	5.23	8.08	8.03	11.15	11.17
Ib	5.44	5.47	8.16	8.20	11.48	11.51

Standard uncertainties, u, are: u(pK_1_) = 0.03, u(pK_2_) = 0.01, u(pK_3_) = 0.02

#### Spectrophotometric studies on Co (II), Ni (II), Cu (II), and Zn (II) complexes with aminopyrazole azo dyes Ia and Ib:

The formation and stoichiometry as well as stability of complexes formed by reaction of Co(II), Ni(II), Cu(II), and Zn(II) with PAP azo- dyes (la and Ib) are studied spectrophotometrically in the visible-near IR region (400–800 nm). The following sections includes a systematic study of the factors affecting the formation of the complexes of Co (II), Ni (II), Cu (II), and Zn (II) with the dyes (la and Ib) and the use of these complexes in the micro-determination of Co(II), Ni(II), Cu(II), and Zn(II) ions.

##### Optimum pH range

**Fig. 5:**
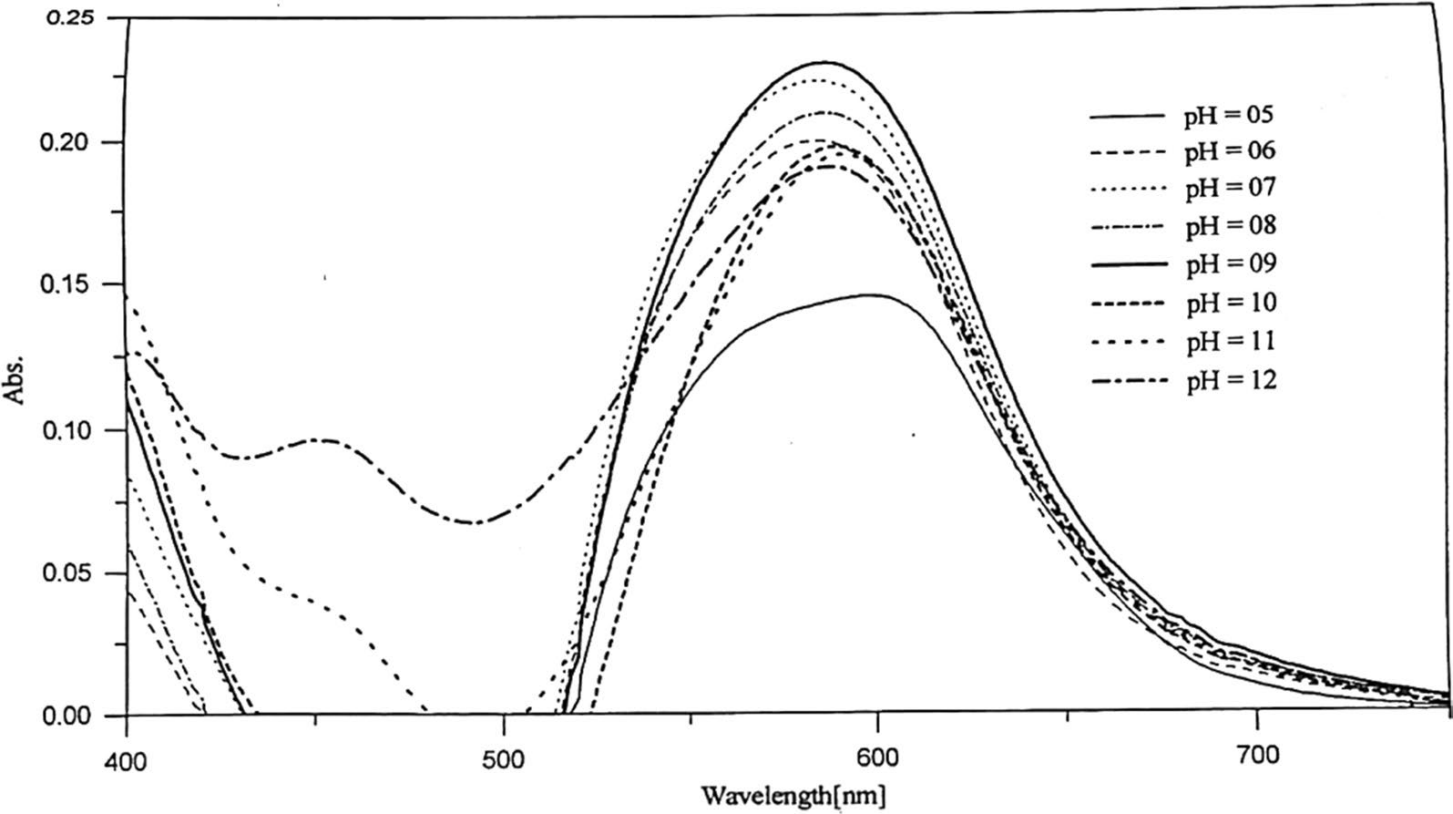
Effect of pH on the absorption spectra of 5 × 10^–5^ mol dm^−3^ of Co (II)-Ia chelate

By studying the absorption spectra of 5 × 10^–5^ mol dm^−3^ Co-Ia complex within the range 800–400 nm at a variable pH range, some conclusions can be outlined. Maximum peak at about 588 nm was observed at the pH's range (2–13), subjected to a slight red shift indicate the complex formation. That behavior is similar when applied to the other metal complexes; [Ni(II), Cu(II), and Zn(II)] of Ia with some changes of the maximum absorbance values, Fig. 5.

**Fig. 6:**
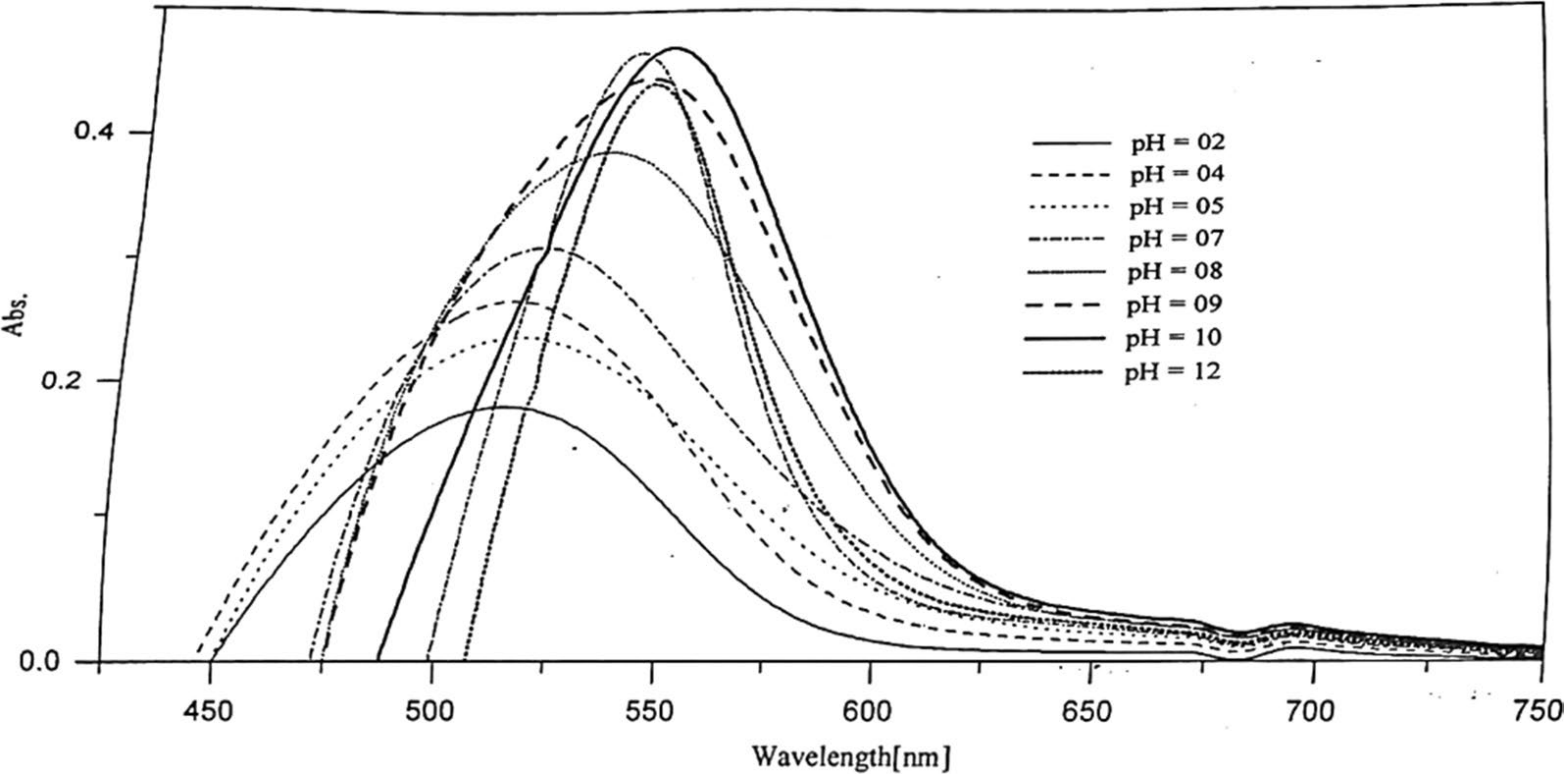
Effect of pH on the absorption spectra of 5 × 10^–5^ mol dm^−3^ of Cu (II)-Ib chelate

Comparing to the Ib-metal ions complexes; taking Cu-Ib complex as example; a slight red shift was observed by rising the pH value, where a maximum absorbance formed at about two regions; band at 510 nm at acidic medium (pH 2–7), while in the alkaline medium (pH 8–13) the band lies at about 545 nm, Fig. 6.

**Fig. 7:**
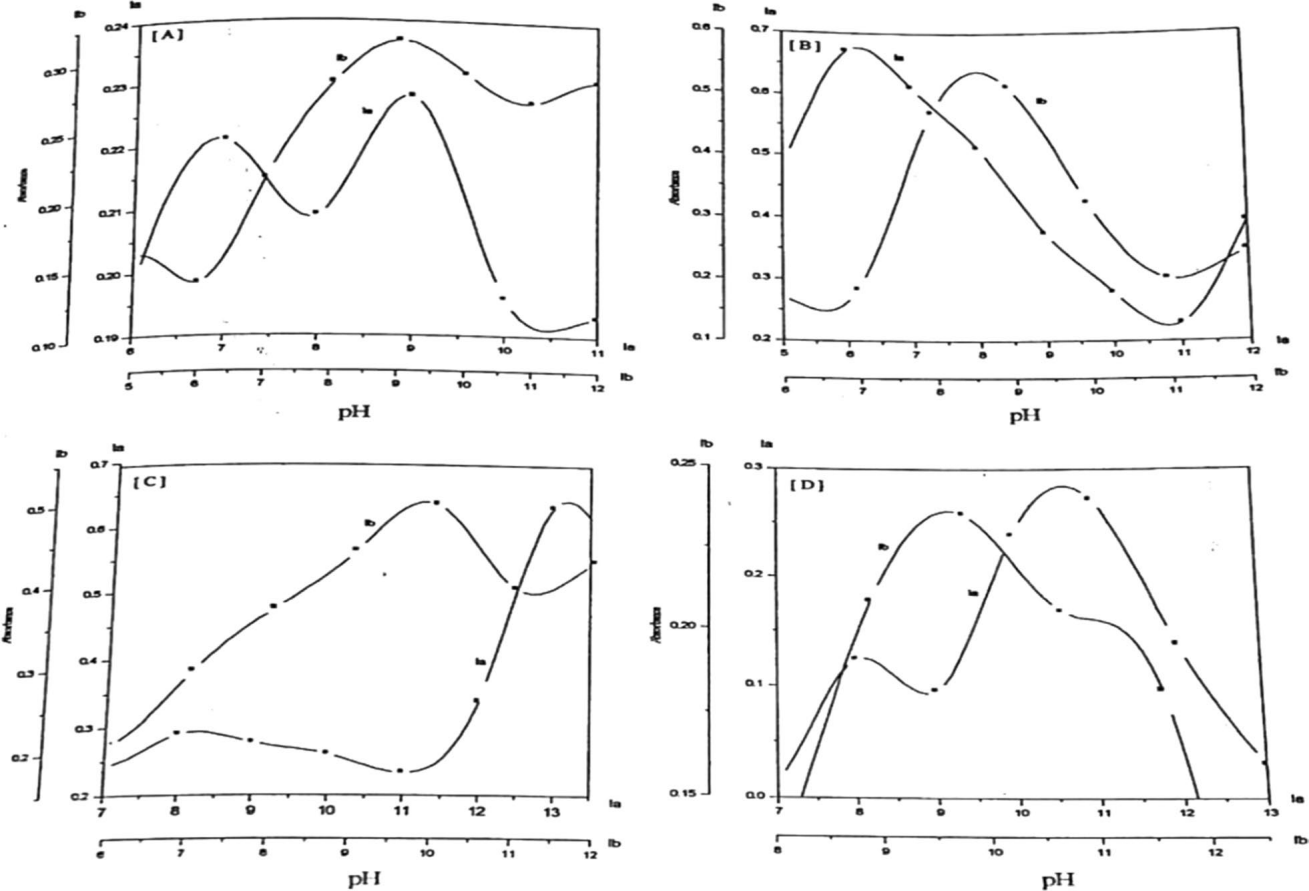
Absorption-pH curves for the chelates of Co(II) (**A**), Ni(II) (**B**), Cu(II) (**C**), and Zn(II) (**D**) with PAP azo-dyes (Ia and Ib)

The most suitable pH for the complex formation is taken from the absorbance-pH plot. Figure 7. The buffer, which gives the highest absorbance value, is thus recommended for subsequent studies, and the color of the formed complexes are given at Table 4.

**Table 4:** Experimental data of the suitable buffer solution for the formation of the PAP azo complexes (Ia and lb)

Ligand	Co(II)		Ni(II)		Cu(II)		Zn(II)	
	Buffer pH	color	Buffer pH	color	Buffer pH	color	Buffer pH	color
Ia	Universal 9	Yellow–green	Universal 6	Pink	Universal 13	Red	Universal 10.5	Bright-red
Ib	Universal 9	Dark red	Universal 9	Orange-Red	Universal 10	Red	Universal 9.5	Orange–red

##### Selection of the suitable wavelength

**Fig. 8:**
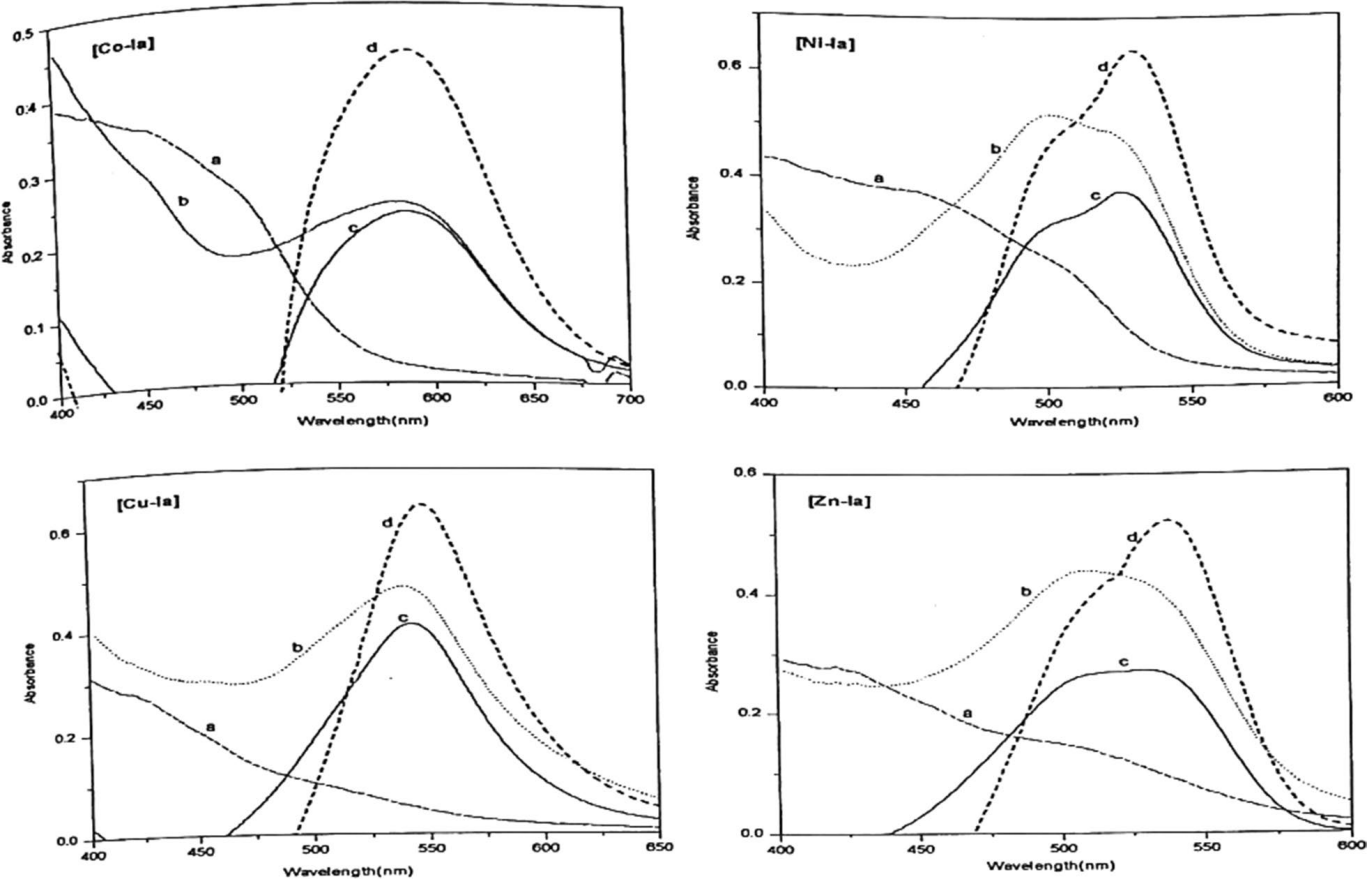
Absorption spectra of PAP azo-dye Ia and their chelates with Co(II), Ni(II), Cu(II), and Zn(II). **a** dye vs.H_2_O **b** Complex vs. H_2_O **c** complex vs. dye 1:1 **d** complex vs. dye 1:2

**Fig. 9:**
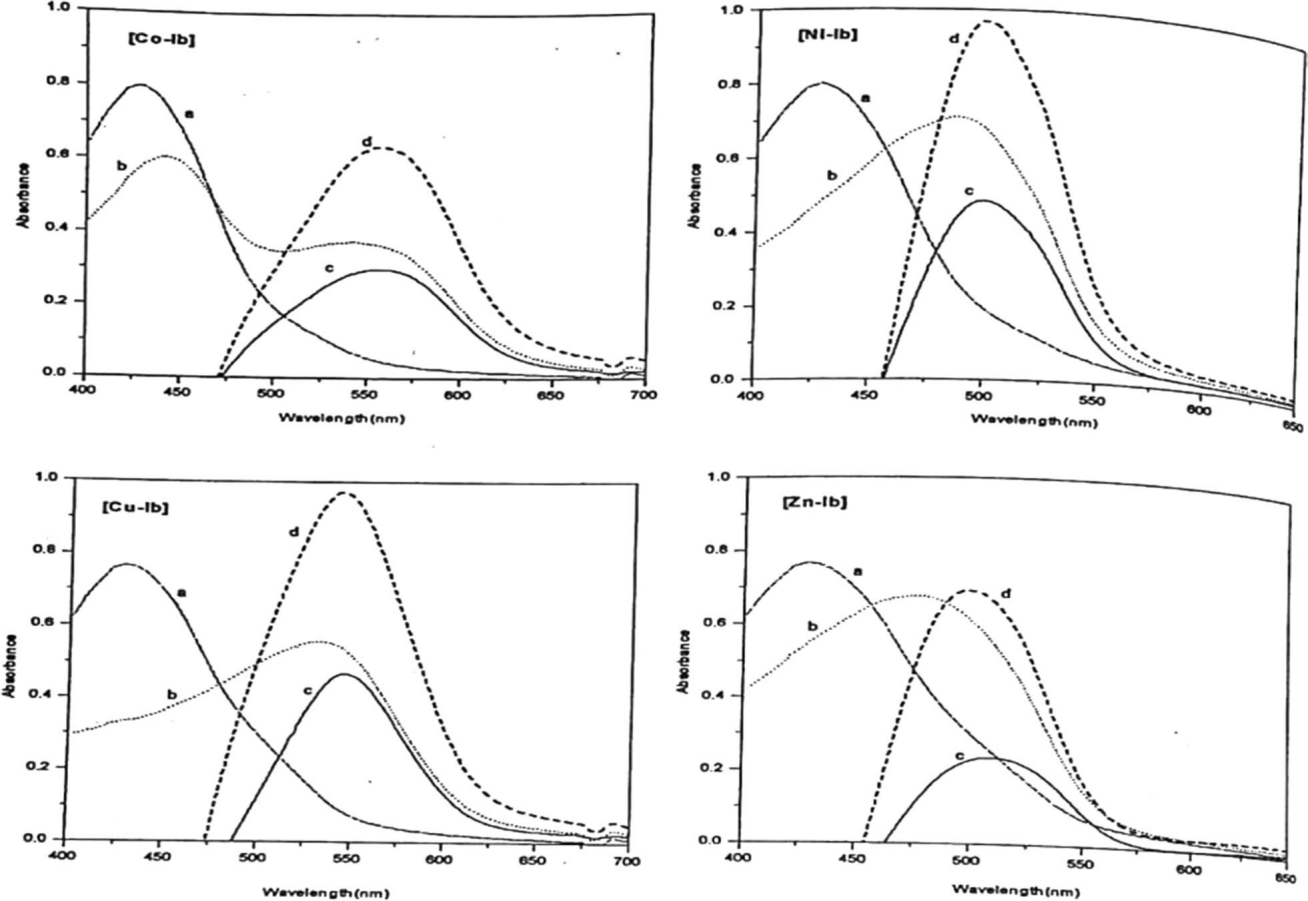
Absorption spectra of PAP azo-dye Ib and their chelates with Co(II), Ni(II), Cu(II), and Zn(II). **a** dye vs.H_2_O **b** Complex vs. H_2_O **c** complex vs. dye 1:1 **d** complex vs. dye 1:2

In the representative Figs. 8 and 9, absorption spectra were recorded in the visible range at the recommended pH value. Table 4, for the dye solution (curve a), the complex solution (curve b), both against water as a blank and the complex solution against the dye, the difference curve, (curve c). From curve (c) the wavelength corresponding to the maximum absorption was determined and chosen as the optimum wavelength for the subsequent measurements. The difference in *λ*_*max*_ (Table 5) of the complex and the dye by amount of 50–140 nm manifests itself also in the difference in color between the dye and the corresponding metal- complex.

**Table 5:** Experimental data of the λ_max_ for the investigated PAP azo dyes and their chelates at the recommended pH values

Dye	λ_nm_				λ_nm_				λ_nm_				λ_nm_			
	a	b	c	Δ λ_nm_	a	b	c	Δ λ_nm_	a	b	c	Δ λ_nm_	a	b	c	Δ λ_nm_
	Co(II)				Ni(II)				Cu(II)				Zn(II)			
Ia	439	580	587	141	430	502	525	72	422	540	544	118	429	510	530	81
Ib	428	540	557	112	428	488	499	60	428	533	546	105	428	477	508	49

^a^wavelength of the dye using water as a blank

^b^wavelength of the chelate using water as blank

^c^wavelength of the chelate using the dye as a blank (1:1)

^d^wavelength of the chelate using the dye as a blank (1:2)

From the spectrophotometric results, it is evident that one of the important factors affecting the stability of the different complexes is the great difference in color between the dye and the corresponding metal- complex. Form the results collected in Table 5 of λ_max_, one can note that the greater the Δλ_max_ value, the higher the stability of the formed complex, In comparing the stability of he formed complexes according to the Δλ_max_, the following sequence was generally observed; Ni(II) < Zn(II) < Cu(II) < Co(II) in the case of Ia complexes, and Zn(II) < Ni(II) < Cu(II) < Co(II) for the Ib complexes.

**Fig. 10:**
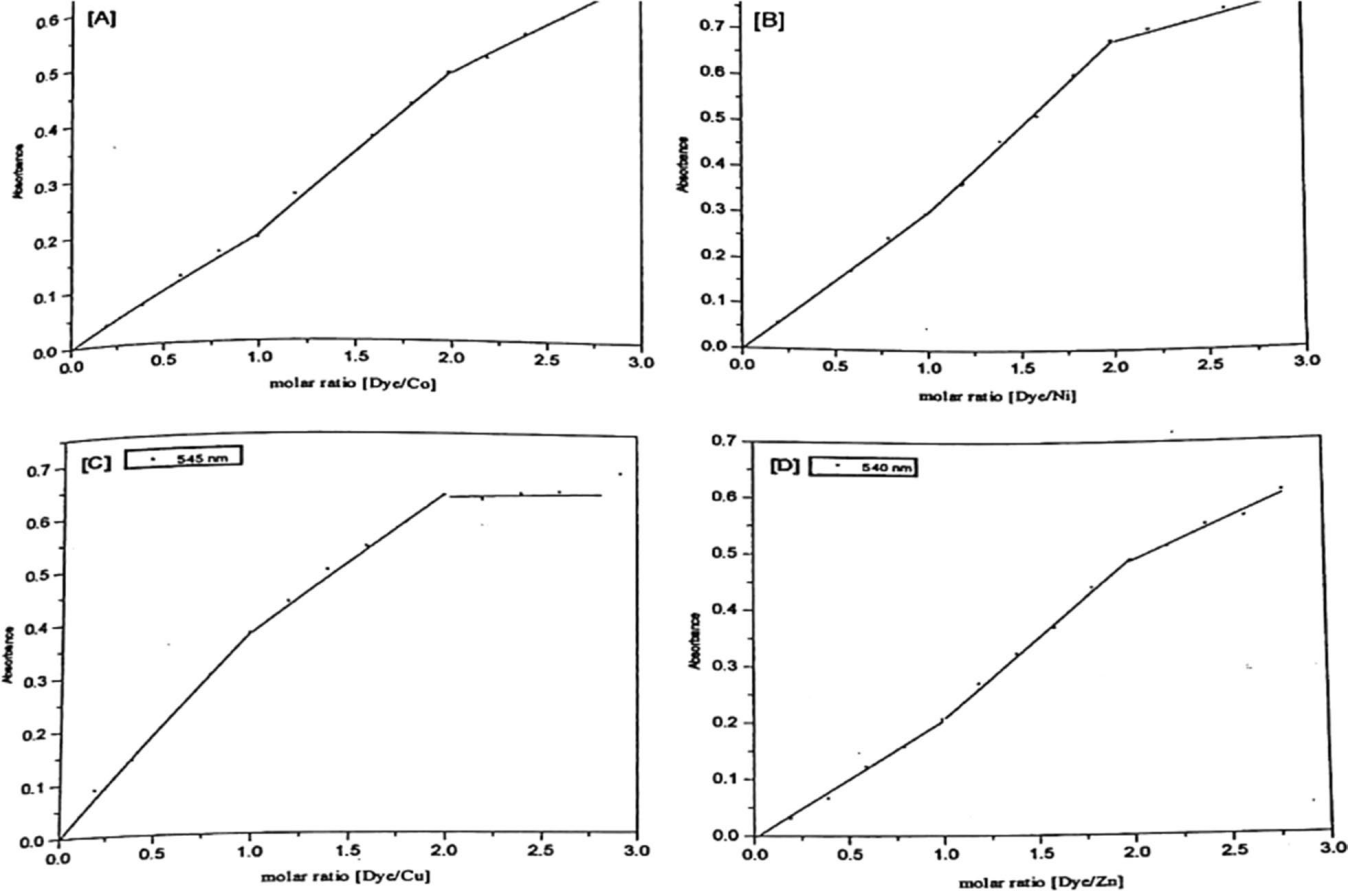
Molar ratio of Co(II) (**A**), Ni(II) (**B**), Cu(II) (**C**), and Zn(II) (**D**) chelates with PAP azo-dye Ia

**Fig. 11:**
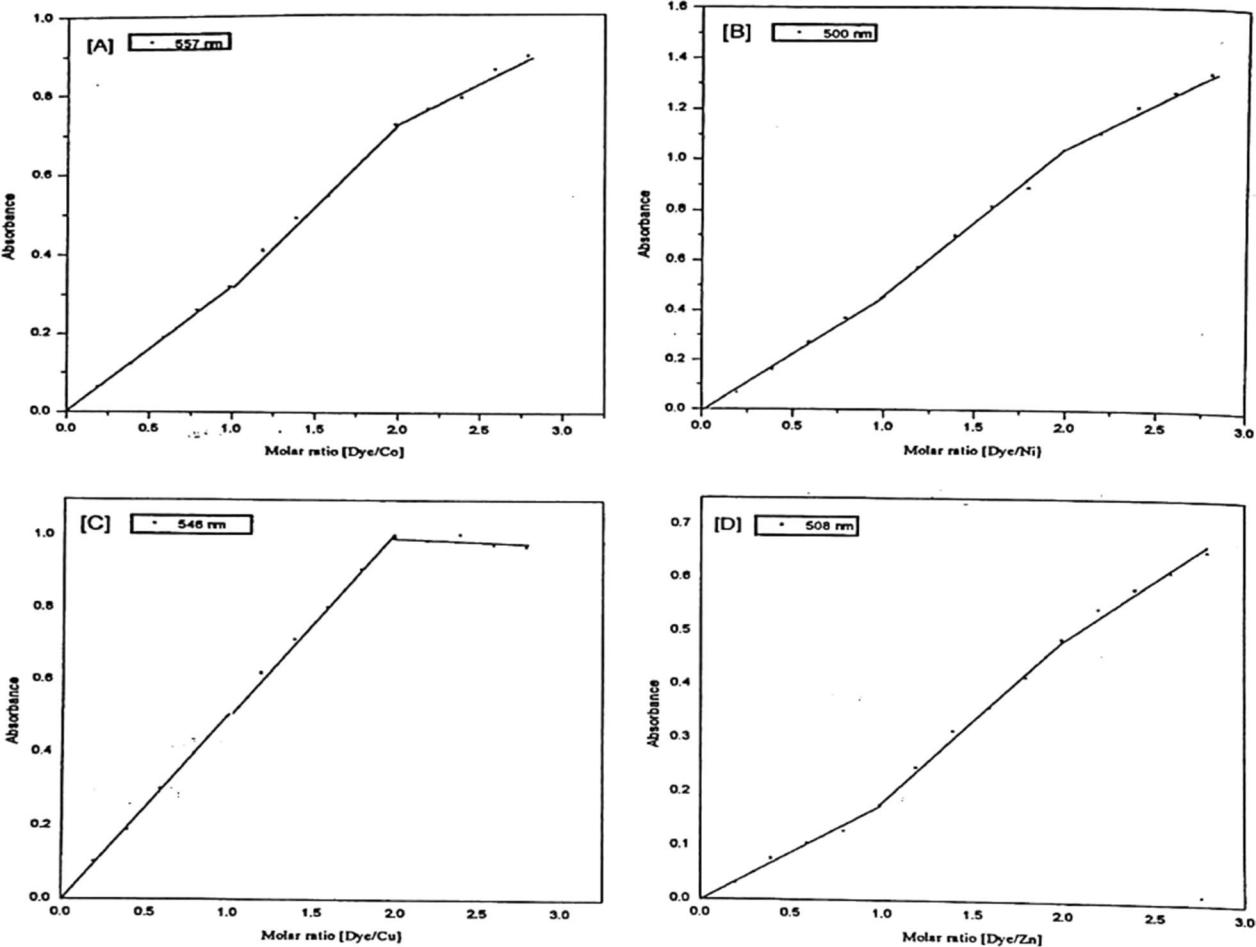
Molar ratio of Co(II) (**A**), Ni(II) (**B**), Cu(II) (**C**), and Zn(II) (**D**) chelates with PAP azo-dye Ib

By mixing 0.5 ml 10^–3^ mol dm^−3^ metal solution with 1 ml 10^–3^ mol dm^−3^ Ia or Ib dyes respectively, and 5 ml of recommended buffer solution and measuring the absorbance against the dye, different curve (d) is obtained for the 1:2 complex. All of the metal ions under investigation form 1:2 complexes as presented in Figs. 10 and 11. The wavelengths at the maximum absorbance (curve c) collected in Table 5 were chosen as the optimum wavelengths for studying the other factors influencing the formation of the metal ion complexes of Ia and Ib.

##### Sensitivity and sequence of addition

Under the stated optimum conditions, it was found that a minimum of 0.5 ppm of Co(II), Cu(II), Ni(II) and Zn(II) could be determined using Ia and Ib with fair accuracy and precision, indicating the sensitivity of the method. The study of three sequence of additions (dye-metal—buffer, dye -buffer- metal, metal -buffer -dye) was done to select the most suitable one for the developing the concerned complexes. It was found that, the sequence (dye- buffer -metal) is the best for the formation of Co (II)-(Ia and Ib) and Cu(II)-Ib complexes, while the sequence (dye -metal – buffer) is the best for the other complexes.

##### Effect of time

The effect of time, on the formation and the stability, of complexes of Cu(II), Co(II), Ni(II) and Zn (II) with Ia and Ib azo dyes, was studied by measuring the absorbance of 5 × 10^–5^ mol dm^−3^ solution against blank solution at the maximum wavelength.

The results show that, the (Cu, Ni, Zn)-Ia and (Cu, Ni, Zn)-Ib complexes are formed instantaneously and remain stable for more than 48 h. While Co-Ia and Co-Ib complexes are stable for about 24 h. The formed complexes show no dissociation after several days but Cu-Ia and Cu-Ib complexes could suffer a precipitation.

##### Effect of organic solvents

The effect of some organic solvents such as methyl alcohol, ethyl alcohol, acetone and DMF (5–35%) on the formation of the investigated complexes at the recommended maximum wavelength characteristic to every complex has been studied. It was found that, in the case of Cu-Ia and Cu-Ib, acetone, methanol and ethanol up to (35% by volume) have a small positive effect on the absorbance while DMF up to (25% by volume) cause a decrease in the absorbance. Also, it was found that methyl alcohol and ethyl alcohol and acetone in all proportions (5–35% by volume) cause slight increase in the absorbance for Co-Ia complexes while DMF causes sharp decreases in the absorbance in all proportions. For Co-Ib complex: methyl alcohol, ethyl alcohol, acetone and DMF in all proportions, decrease the absorbance.

In the case of Ni-Ia complex methyl alcohol, ethyl alcohol and acetone increase the absorbance while DMF, in all proportions, decreases the absorbance. For Ni-Ib complex methyl alcohol, ethyl alcohol and acetone, in all proportions, decrease the absorbance while DMF increase absorbance. Finally, it was found that the absorbance for Zn-Ia complex suffers a slight increase in the presence of methyl alcohol, ethyl alcohol and acetone while it suffers a sharp decrease in DMF. For Zn-Ib complex, acetone and DMF decrease the absorbance, while methyl alcohol and ethyl alcohol cause an increase in the absorbance in all proportions. We should mention that, the presence of such organic solvents is not recommended in the developing of such complexes for analytical purposes, but we shouldn't forget that the two reagents Ia, and Ib dissolved in ethyl alcohol and as we stated before, all measures were at mixed solvent methanol–water (5% by volume).

##### Influence of foreign ions

A systematic quantitative study was carried out by measuring the absorbance of a solution containing 32 g$\mu$ of metal ion [Co(II), Ni(II), Cu(II), or Zn(II)] and 2, 10, 20 folds, as metal, of variety of cations and anions, 1 ml 10^–3^ mol dm^−3^ dye, 5 ml universal buffer characteristic to every metal ion, and twice distilled water up to 10 ml. The results indicate that the ions were considered interfering when an increase in the absorbance by 5% of its initial value (in the absence of non-interfering ions) was observed, while those showing no changes in the absorbance are considered non-interfering. The interfering and non-interfering ions in the determination of Co(II), Ni(II), Cu(II) and Zn(II) complexes are listed in Table 6 with the recommendation that the interfering ions must be absent in Co(II), Ni(II), Cu(II) and Zn(II) determination.

**Table 6:** Experimental data of the influence of foreign ions on the determination of Co (II), Ni(II), Cu(II) and Zn(II) complexes with the investigated PAP azo dyes Ia and Ib

Foreign ions	Co(II)		Ni(II)		Cu(II)		Zn(II)	
	Ia	Ib	Ia	Ib	Ia	Ib	Ia	Ib
Na^+^	−	−	−	−	−	−	−	−
Mn^2+^	−	−	−	−	−	−	+	+
Mg^2+^	−	−	−	−	−	−	−	−
Fe^3+^	+	+	+	+	+	+	+	+
Ca^2+^	−	−	−	−	−	−	−	−
Pb^2+^	+	−	−	−	−	−	+	−
Zn^2+^	+	+	+	+	+	+		
Ni^2+^	+	+			+	+	+	+
Cu^2+^	+	+	+	+			+	+
Co^2+^			+	+	+	+	+	+
Cd^2+^	+	−	−	−	−	−	+	+
K^+^	−	−	−	−	−	−	−	−
Ba^2+^	−	−	−	−	−	−	−	−
${NO}_{3}^{-}$	−	−	−	−	−	−	−	−
${so}_{4}^{2-}$	−	−	−	−	−	−	−	−
Cl^−^	−	−	−	−	−	−	−	−
I^−^	−	−	−	−	−	−	−	−
Oxalate	−	−	−	−	−	−	−	−
Citrate	−	−	−	−	−	−	−	−
Tartarate	−	−	−	−	−	−	−	−
EDTA	+	+	+	+	+	+	+	+
Al^3+^	+	+	+	+	+	+	+	+
Borate	−	−	−	−	−	−	−	−
Acetate	−	−	−	−	−	−	−	−
Benzoate	+	+	+	+	+	+	−	−

In the case of Co(II) complexes, up to 20 folds of Na^+^, Mn^++^, Mg^++^, Ca^++^,K^+^, Ba^++^, NO_3_^−^, SO_4_^−−^, Cl^−^,I^−^, oxalate, citrate, tartarate, borate, acetate don't interfere. On the other hand Fe^++^, Cu^++^, Zn^++^, Ni^++^, EDTA, benzoate interfere and shouldn't be present, while Pb^++^ and Cd^++^ interfere with Co(II) -Ia, Table 6.

In the case of Ni(II) complexes, up to 20 folds of Na^+^, Mn^++^, Mg^++^, Ca^++^, Pb^++^, Cd^++^, K^+^, Ba^++^, NO_3_^−^, SO_4_^−−^, Cl^−^, I^−^, oxalate, citrate, tartarate, borate, and acetate don't interfere. On the other hand Fe^++^, Co^++^, Zn^++^, Cu^++^, EDTA, and benzoate interfere and shouldn't be present, Table 6.

In the case of Cu(II) complexes, up to 20 folds of Na^+^, Mn^++^, Mg^++^, Ca^++^, Pb^++^, Cd^++^, K^+^, Ba^++^, NO_3_^−^, SO_4_^−−^, Cl^−^, I^−^, oxalate, citrate, tartarate, borate, and acetate don't interfere. On the other hand Fe^++^, Co^++^, Zn^++^, Ni^++^, EDTA, and benzoate interfere and shouldn't be present, Table 6.

In the case of Zn(II) complexes, up to 20 folds of Na^+^, Mg^++^,Ca^++^,Pb^++^,Cd^++^, K^+^, Ba^++^ NO_3_^−^, SO_4_^−−^, Cl^−^, I^−^, oxalate, citrate, tartarate, borate, and acetate, benzoate don't interfere. On the other hand Mn^++^, Fe^++^, Co^++^, Zn^++^, Ni^++^, and EDTA interfere and shouldn't be present while Pb^++^ interfere with Zn (II)-la, Table 6.

##### Molecular ratio of the complexes

The composition and the stability constants of complexes are determined by the following spectrophotometric methods.

###### a. The molar ratio method (MRM) [19]

For the investigation of the molecular structure of the complexes formed in solution, the results obtained from the molar ratio method are represented graphically as absorbance-molar ratio plots, Figs. 10 and 11. The straight lines obtained intersect at the molar ratio of chelates liable to be formed under the above-mentioned conditions. The molar ratio of the complexes that deduced from the absorbance- molar ratio plots is listed in Table 7.

**Table 7:** Experimental data of the **s**toichiometry (M: L) of Co (II), Ni(II), Cu(II) PAP azo complexes (Ia and Ib)

Dye	Co(II)		Ni(II)		Cu(II)		Zn(II)	
	MRM	CVM	MRM	CVM	MRM	CVM	MRM	CVM
Ia	1:1	1:1	1:1	1:1	1:1	–	1:1	1:1
	1:2	1:3	1:2	1:2	1:2	1:2	1:2	1:2
Ib	1:1	–	1:1	–	–	1:1	1:1	1:1
	1:2	1:3	1:2	1:3	1:2	1:2	1:2	1:2

In the case of Cu (II), the absorbance of these solutions was measured at 545 nm for Cu-Ia and 546 nm for Cu-lb complexes.The plot is composed of three linear portions intersecting at the molar ratio 1:1 and 1:2 (Cu: ligand) in the case of Cu-la and is represented by a plot composed of two linear portions intersecting at the molar ratio 1:2 (Cu: ligand) chelate in the case of Cu-Ib.

For Co (ll), the absorbance of these solutions was measured at 590 nm for Co-la and 557 nm for Co-Ib complexes, respectively. The absorbance- molar ratio relationship is represented by the curve composed of three linear portions intersecting at the molar ratio 1:1 and 1:2 (Co: ligand) chelate in the case of the two complexes Co-la and also Co-Ib.

In the case of Ni (II), the absorbance of these solutions was measured at 530 nm for Ni-la and 500 nm for Ni-Ib complexes, respectively. The absorbance molar ratio relationship is assigned by a curve consisting of three linear portions which intersect at the molar ratio 1:1, 1:2 (Ni: ligand) chelate in the case of Ni-la complex, and for Ni-Ib complexes a curve composed of three linear portions intersecting at the molar ratio 1:1 and 1:2 (Co: ligand) complexes which may take as evident for the existence of both 1:1 and 1:2 Ni-Ib complexes in the solution.

In the case of Zn (II), the absorbance of these solutions was measured at 540 nm for Zn-la and 508 nm for Zn-Ib complexes, respectively. The absorbance-molar ratio relationship is represented by a curve composed of three linear portions intersecting at the molar ratio 1:1 and 1:2 (Zn: ligand) in the case of Zn-la and Zn-Ib complexes, Figs. 10 and 11.

###### b. The continuous variation method (CVM) [20, 21]

**Fig. 12:**
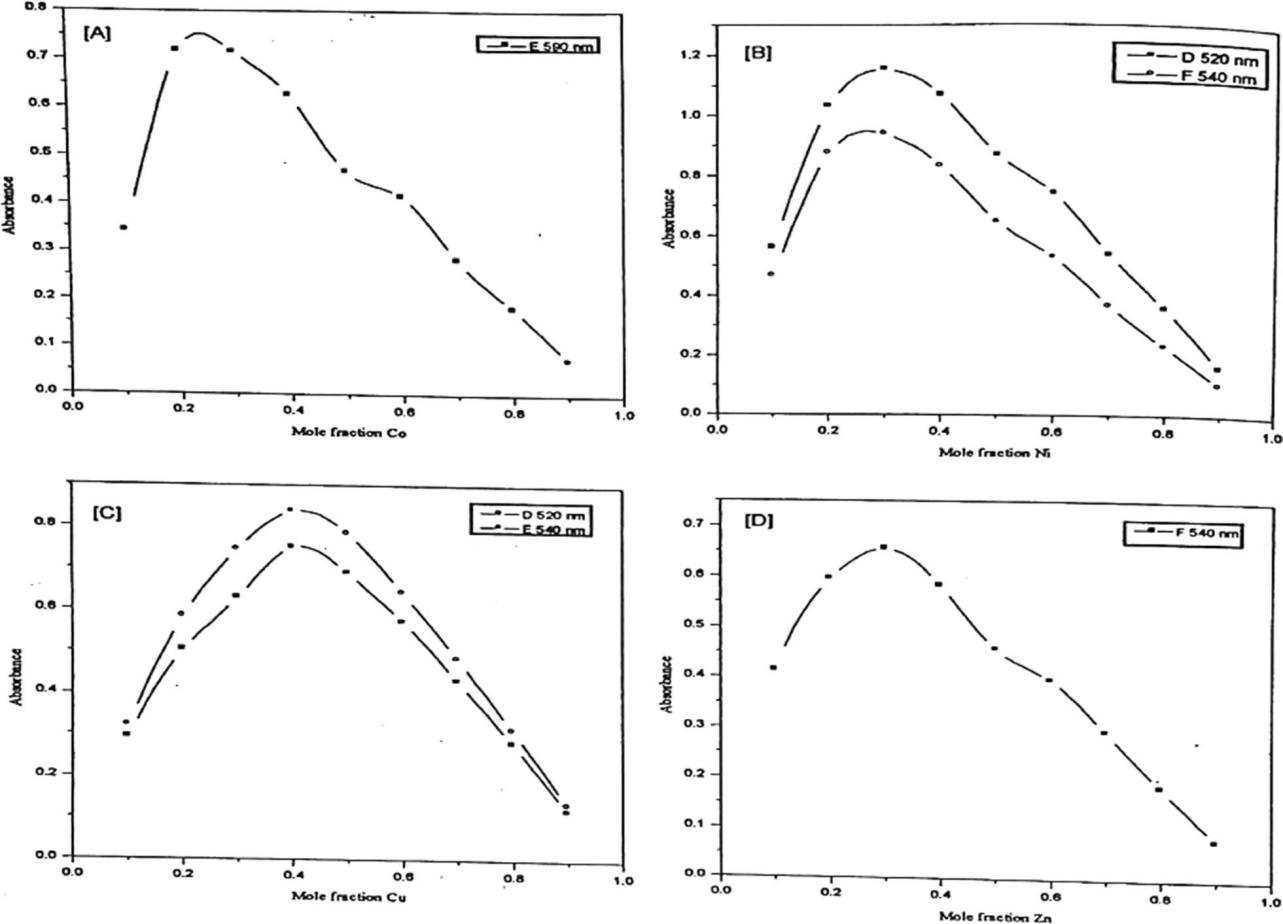
Continuous variation plots of Co(II) (**A**), Ni(II) (**B**), Cu(II) (**C**), and Zn(II) (**D**) chelates with PAP azo-dye Ia

**Fig. 13:**
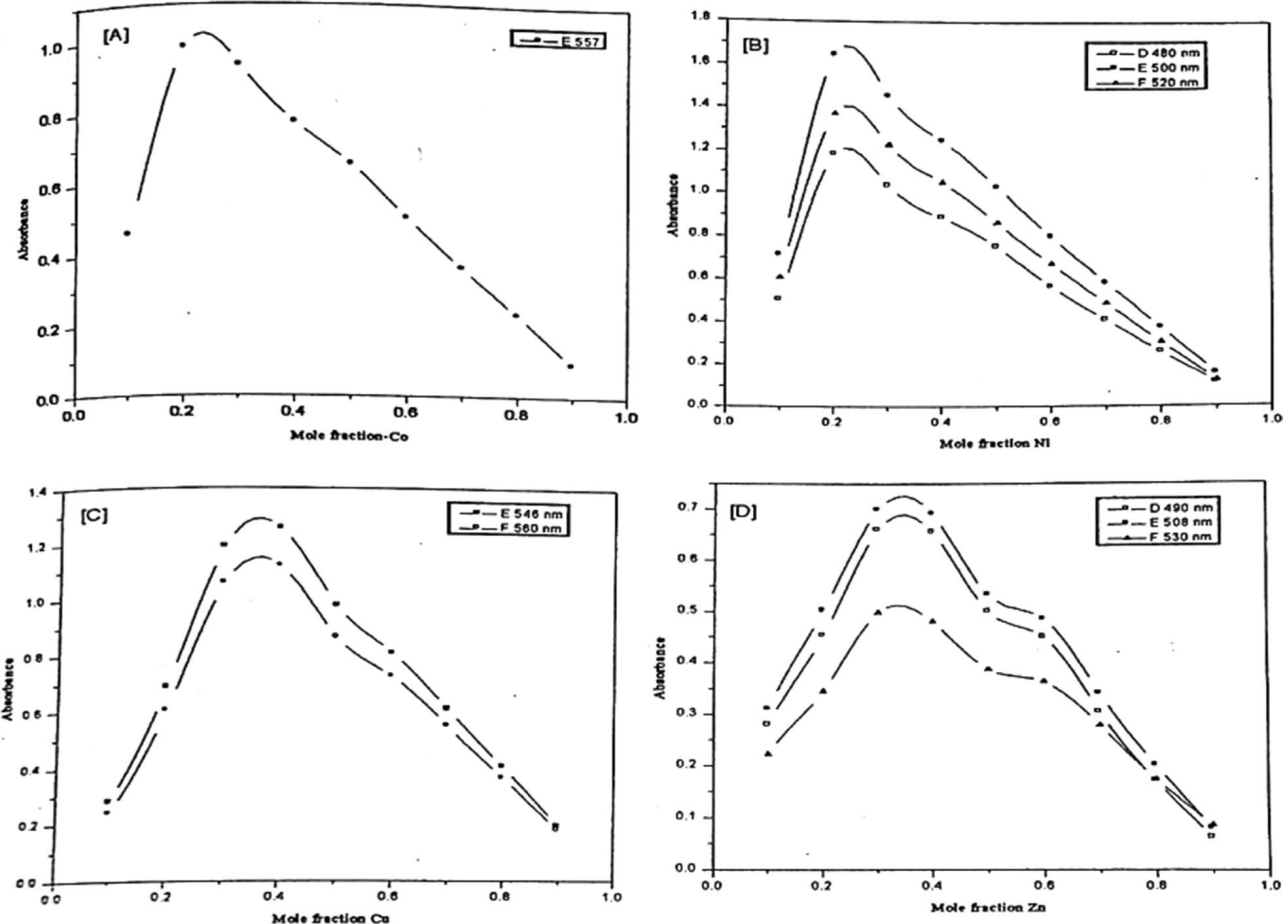
Continuous variation plots of Co(II) (**A**), Ni(II) (**B**), Cu(II) (**C**), and Zn(II) (**D**) chelates with PAP azo-dye Ib

Representative plots of the absorbance versus the mole-fraction of the metal ion under investigation are given in Figs. 12 and 13. The curves obtained are characterized by the presence of maxima at the stoichiometric ratio of the complex formed. The molecular ratios of the complexes under investigation obtained from the CVM are listed in Table 7.

In the case of Cu (II), the absorption spectra of the prepared solutions were measured at wavelengths 520,530 and 540 nm. For Cu-Ia chelate the absorption curves show a maximum at Cu mole fraction of 0.4, providing evidence that a 1:2 complex is formed in such solution, for Cu-Ib complex the absorption curve show a maximum at Cu mole fraction of 0.35 and a shoulder at 0.5 indicating that a 1:2 and 1:1 complexes are formed in such solutions.

In the case of Co (II), the absorption spectra were recorded for such solutions at wavelengths 590 and 557 nm. For Co-Ia complex the absorption curves show a maximum at Co mole fraction of 0.25 and a shoulder at 0.5, providing evidence that a 1:3 and 1:1 complexes are formed in such solutions, for Co-Ib complex the absorption curve show a maximum at Co mole fraction 0.25 indicates that a 1:3 complex is formed in such solutions with a contribution of 1:1 complex which may be formed under such conditions.

In the case of Ni (II), the absorption spectra were measured at absorbance maxima 520 and 540 nm for Ni-la chelate and 480,500,520 nm for Ni-Ib complexes. For Ni-Ia complex the absorption spectra show a maximum at Ni mole fraction of 0.3 and a shoulder at 0.5, indicating that a 1:2 and 1:1 complexes are provided in such solutions, for Ni-Ib complex the absorption curve show a maximum at Ni mole fraction 0.25 give a conclusion that 1:3 complex could be formed at that maintained conditions.

For Zn (II), the absorption spectra were recorded for such solutions at wavelengths 520 and 540 nm for Zn-Ia chelate and 490,508,530 nm for Zn-lb complexes. The absorption spectra show a maximum at Zn mole fraction of 0.3 and 0.35 for Zn-la and Zn-Ib respectively, providing evidence that a 1:2 and 1:1 complex were formed in such solutions at those maintained conditions.

**Scheme 2:**
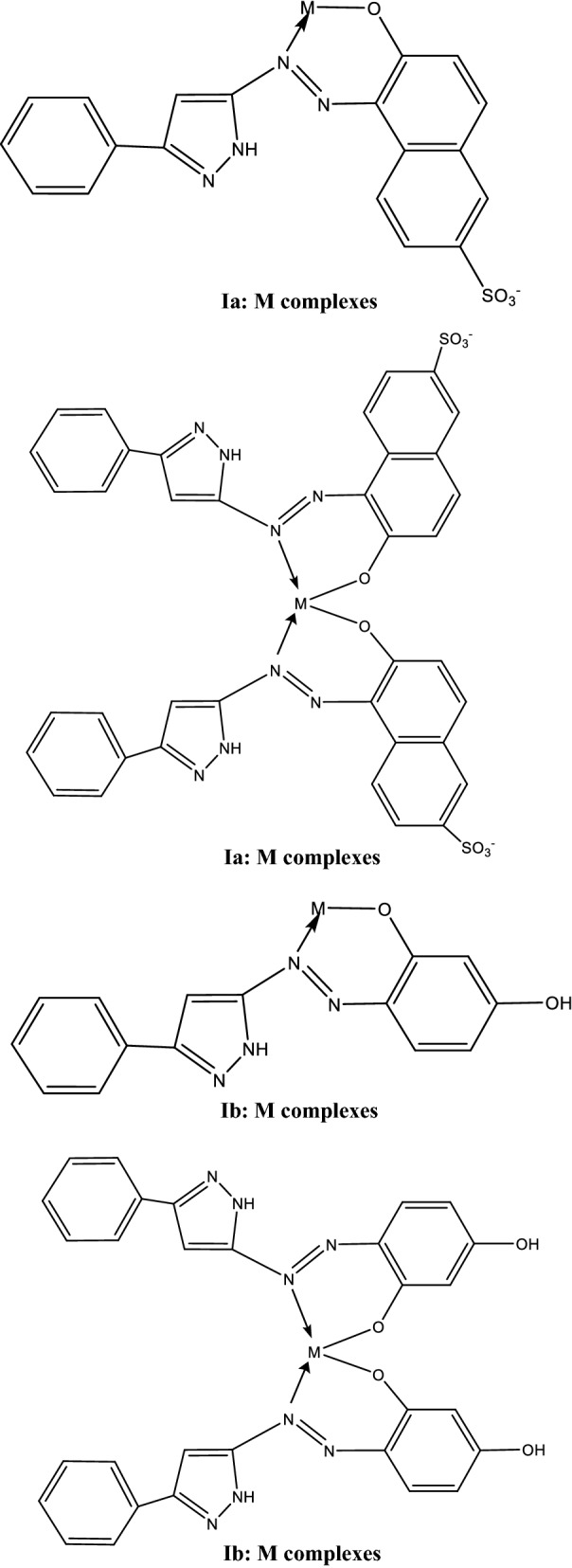
The suggested structure of the formed stoichiometric complexes

The suggested structure of the formed stoichiometric complexes was represented in Scheme 2.

##### Obeyance of Beer’s law

**Fig. 14:**
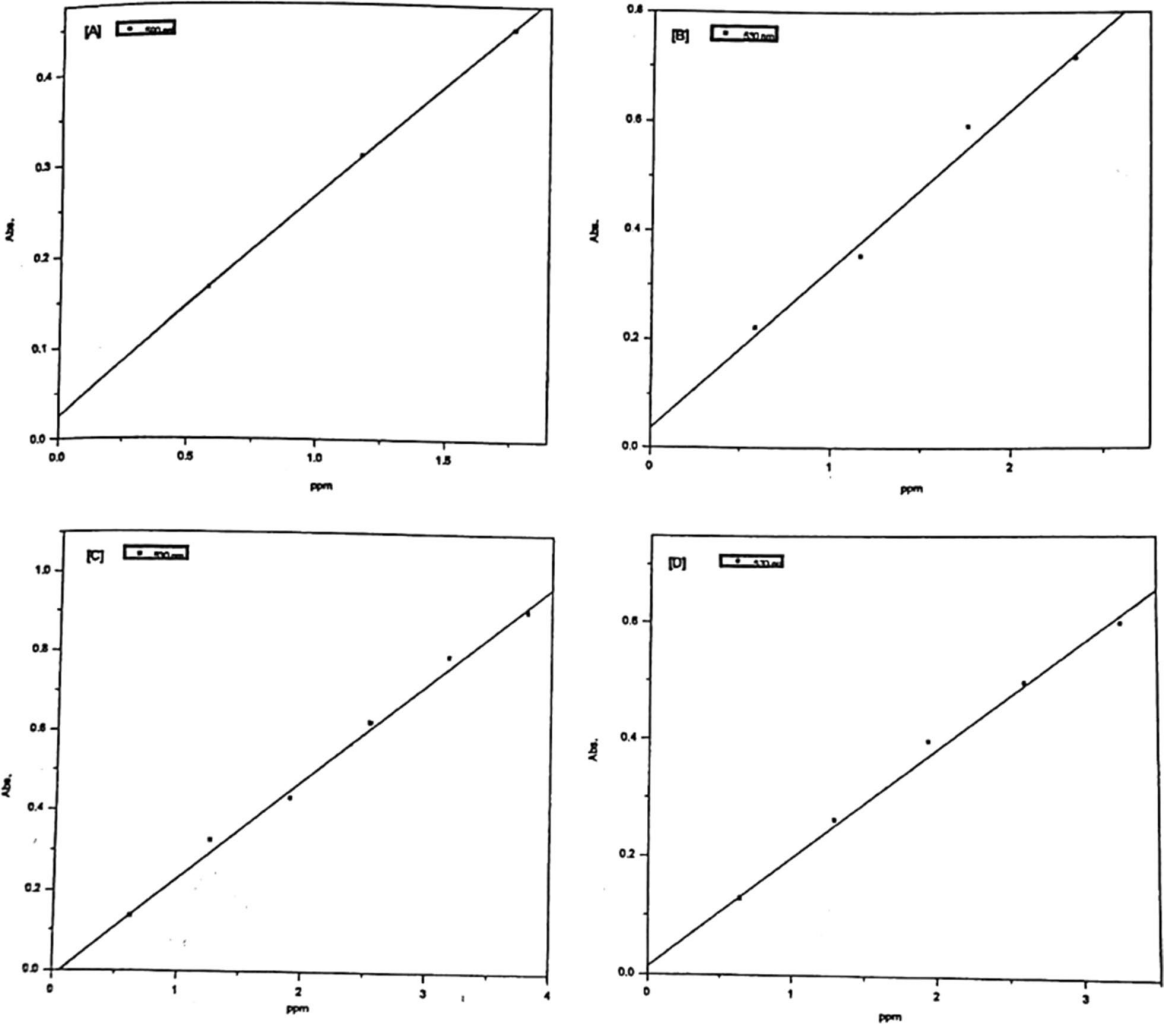
Validity of Beers law for Co(II) (**A**), Ni(II) (**B**), Cu(II) (**C**), and Zn(II) (**D**) chelates with PAP azo-dye Ia

**Fig. 15:**
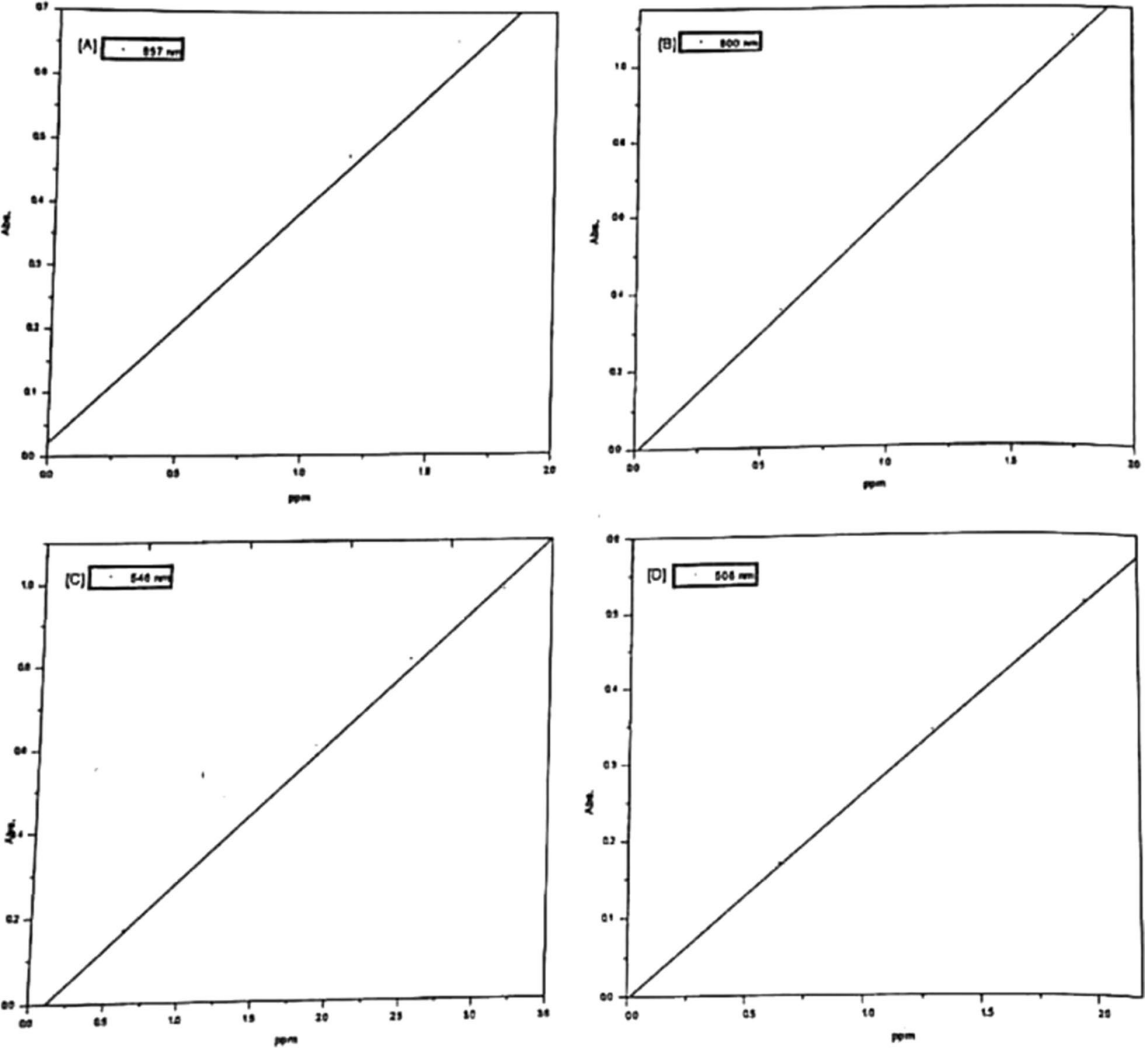
Validity of Beers law for Co(II) (**A**), Ni(II) (**B**), Cu(II) (**C**), and Zn(II) (**D**) chelates with PAP azo-dye Ib

The obeyance of Cu (II), Ni (II), Co (II) and Zn (II) complexes with the dyes under investigation to beer's law was verified in order to use such chelates for the spectrophotometric determination of Cu (II), Ni (II), Co (II) and Zn (II) ions. Representative plots are given in Figs. 14 and 15. The absorbance’s of Cu (II), Ni (II), Co (II) and Zn (II) complex solutions were measured at the recommended wavelengths and pH values applying the optimum blank compensation method [25], in which the amount of unreacted ligand is used as a blank, since the dyes have appreciable absorbance at these wavelengths.

The molar absorptivity values ($\varepsilon$) expressed in l. mol^−1^.cm^−1^ are obtained as the slopes of the constructed lines from which the specific absorptivity (a) (ml.g^−1^.cm^−1^) [26], corresponding to the absorbance of 1 g$\mu$/ml in a cuvette with an optical pathlength of 1 cm, and Sandell's sensitivity (S) [27]; $\mu$ g/cm^−2^ which represents the number of micrograms of the determinate per of cm^3^ solution having an absorbance 0.001 for pathlength of 1 cm are calculated. All these values are treated statistically and the correlation coefficient and standard deviation are calculated and summarized in Table 8. From Table 8, it is clear that Sandell sensitivity values are small, therefore, the use of optimum blank compensation technique increases the sensitivity of the present method.

**Table 8:** Experimental data of the molar absorptivity (ε), Specific absorptivity [17] (**a**), Sandell's sensitivity [18] (S), lowest detectable concentration (LDC) and statistical results for Ia and Ib and other literature chelates

Chelate	Metal	$\varepsilon$ × 10^–4^ l. mol^−1^.cm^−1^	a ml.g^−1^.cm^−1^	I	s	R^2^	S $\times$ 10^–3^ $\mu$ g/cm^2^	$\mu g/10$ ml up to	LDC, ppm
Ia	Co(II)	1.49	0.25	0.027	0.012	0.9981	3.59	18.7	0.50
	Ni (II)	1.88	0.32	0.036	0.035	0.9921	3.13	25.0	
	Cu(II)	1.55	0.24	− 0.015	0.024	0.9970	4.17	40.0	
	Zn(II)	1.20	0.18	0.014	0.023	0.9950	5.47	35.0	
Ib	Co(II)	2.25	0.38	0.022	0.018	0.9981	2.61	18.5	0.50
	Ni (II)	3.60	0.55	− 0.008	0.010	0.9991	1.80	18.9	
	Cu(II)	2.04	0.31	− 0.036	0.020	0.9980	3.10	35.0	
	Zn(II)	1.73	0.26	− 0.002	7 × 10^–4^	1.0000	3.80	22.0	
4-(2-pyridylazo) resorcinol (PAR) [2]	Co(II)	–	–	0.069	–	0.9985	–	–	–
	Ni (II)	**–**	–	0.071	–	0.9910	–	–	–
	Cu(II)	**–**	–	0.075	–	0.9960	–	–	–
	Zn(II)	**–**	–	0.080	–	0.9995	–	–	–
2-Carboxy-2′-hydroxy-5′-sulfoformazyl-benzene (Zincon) [2]	Co(II)	**–**	–	0.045	–	0.9870	–	–	–
	Ni (II)	**–**	–	0.046	–	0.9988	–	–	–
	Cu(II)	**–**	–	0.055	–	0.9987	–	–	–
	Zn(II)	**–**	–	0.051	–	0.9994	–	–	–
2-(4-biphenyl) Imidazo[1,2-Pyrimidine-3-Hydrazone [3]	Cu(II)	0.0175	–	0.060	–	0.9909	2.78 × 10^3^	–	0.122
4-(2-pyridylazo) resorcinol (PAR) [1]	Co(II)	–	–	–	–	–	–	–	0.59
dimethylglyoxime (DMG) [1]	Ni (II)	–	–	–	–	–	–	–	5.87
bathocuproine (Bc) [1]	Cu(II)	–	–	–	–	–	–	–	0.32
Extended Kalman filter spectrophotometry [4]	Cu(II)	–	–	0.284	–	0.9964	–	–	0.50
	Co(II)	–	–	-0.144	–	0.9987	–	–	0.30

R^2^: Correlation coefficient. s: Standard deviation. I: Intercept

In the case of the determination of Cu (II), the curves indicate that the variation of the absorbance with concentration is linear up to 6 × 10^–5^ mol dm^−3^ Cu (II) and 5 × 10^–5^ mol dm^−3^ for Ia and Ib respectively. These high values indicate that such procedure is sensitive. By the aid of the Cu-Ia and Cu-Ib, Cu up to 3.8 ppm and 3.18 ppm respectively can be determined spectrophotometrically with requisite accuracy.

In the case of the determination of Co (II), the curves indicate that the variation of the absorbance with concentration is linear up to 3 × 10^–5^ mol dm^−3^ Co (II) for Ia and Ib, and that such a variation obeys beer's law.. By the aid of the Co-la and Co-Ib, Co up to 1.77 ppm can be determined spectrophotometrically with requisite accuracy.

In the case of Ni (II), the curves indicate that the variation of the absorbance with concentration is linear up to 3 × 10^–5^ mol dm^−3^ Ni (II) for la and Ib, and that such a variation obey beer's law. By the aid of the Ni-Ia and Ni-Ib, Ni up to 1.76 ppm can be determined spectrophotometrically with requisite accuracy.

In the case of the determination of Zn (II), the curves indicate that the variation of the absorbance with concentration is linear up to 5 × 10^–5^ mol dm^−3^ Zn (II) and 4 × 10^–5^ mol dm^−3^ for la and Ib, respectively. These high values indicate that such procedure is sensitive. By the aid of the Zn-la and Zn-Ib, Zn up to 2.61 ppm and 1.96 ppm respectively can be determined spectrophotometrically with requisite accuracy.

The obtained data in the present work were compared with that in literatures for the spectrophotometric determination of Cu (II), Ni (II), Co (II) and Zn (II) using another chelates. The literature values are presented in Table 8.

Inspection of the data in Table 8, one can note that there are some chalets; {4-(2-pyridylazo) resorcinol}(PAR) for Co (II) (0.50 ppm) [1] (59 ppm) and Extended Kalman filter spectrophotometry for Cu (II) (0.50 ppm) [4] (0.50 ppm) have comparable lowest detectable concentration (LDC) values with the chalets under study. On the other hands, some chalets {2-(4-biphenyl) Imidazo[1,2-Pyrimidine-3-Hydrazone for Cu (II) [3] (0.122 ppm), bathocuproine (Bc) for Cu (II) [1] (0.32 ppm) and {Extended Kalman filter spectrophotometry for Co (II) [4](0.30 ppm) have best values than that of the chalets under study. Dimethylglyoxime (DMG) [1] have less sensitivity (higher (LDC), 5.87 ppm) value) than that of the chalets under study (0.50 ppm) for Co (II).

##### Procedure for the spectrophotometric determination of Cu (II) using PAP azo dyes (Ia and Ib) as indicators

The following procedure is recommended for the determination of Cu (II) using 2-(3^‛^-phenylpyrazol-5^‛^-yl azo) Schäffer acid (la) and 2-(3^‛^- phenylpyrazol-5^‛^ -yl azo) resorcinol (Ib). To a solution containing up to 38 and 31.8 g$\mu$ of Cu in a 10 ml volumetric flask, 2 ml 10^–3^ mol dm^−3^ dye solution were added and made up to the volume with the recommended buffer (pH 13 and 10). The solution was mixed well and the absorbance was measured at 540 nm against a blank containing the same ingredients except Cu ion using the optimum blank compensation method [28]. The concentration was computed by extrapolation from the calibration curve prepared in the same manner. The standard deviation for determining 12, and 8 g$\mu$ of Cu using Ia and Ib respectively (10 determinations) amounted to 0.03 indicating the reproducibility of the present method. The present method is rapid, simple, sensitive and accurate for the determination of microgram amounts of Co (II), Ni (II), Cu (II) and Zn (II).

##### Spectrophotometric titration of the metal ions with EDTA using (Ia) and (Ib) as indicators

**Fig. 16:**
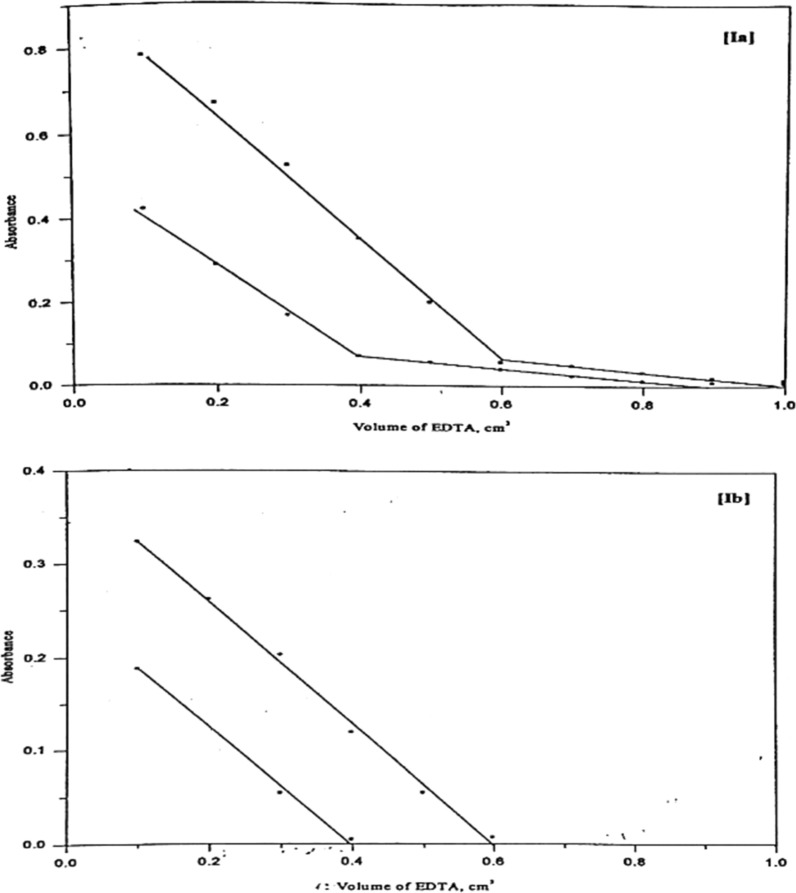
Spectrophotometric titration curves of Ni(II) with EDTA using Ia and Ib azo-dyes

The applicability of Ia and Ib as indicators for the spectrophotometric titration of Ni (II) with EDTA was studied, and the representative curves are shown in Fig. 16. The results of spectrophotometric titration are listed in Table 9. Increasing volumes of EDTA were added to nickel solution, followed by Ia and Ib solutions and universal buffer of the recommended pH values. The absorbance was measured at 530 and 500 nm and plotted against the volume of EDTA. The amount of Ni (II) was calculated from the intersection of the two straight lines obtained.

**Table 9:** Experimental data of the spectrophotometric titration of Ni(II) using Ia and Ib as indicators

Dye	Metal ion amount ($\mu g$)		Recovery %	Recovery error (pph)	Rel.St.Dev %
	Taken	Found^(*)^			
Ia	23.48	23.30	99.23	− 0.77	1.43
	35.22	35.33	100.30	+ 0.30	1.38
Ib	23.48	23.42	99.74	− 0.26	1.21
	35.22	35.16	99.83	− 0.17	0.92

*Mean value of three determinations

The results indicate that the metal ion under investigation can be successfully determined up to 3.52 $\mu$ g with Ia and Ib. Titration curves shown in Fig. 16, indicate that the end point inflections are quite sharp with the applied reagent and hence it can be used as indicator for the spectrophotometric titration of this metal ion with EDTA.

##### Evaluation of the apparent formation constant of Co (II), Ni (II), Cu (II) and Zn (II)-aminopyrazole azo dye chelates from Spectrophotometric results.

The stability constants, Bn, of the formed complexes were calculated using MR and CV methods by the aid of the following Eq. [29]

$$\beta_{n} = \left( {A/A_{m} } \right)/\left[ {1 - \left( {A/A_{m} } \right)} \right]^{n + 1} \left[ L \right]^{n} {\text{n}}^{2}$$

where:

A = absorbance at a given dye concentration, [L].

A_m_ = limiting absorbance.

n = stoichiometric ratio (number of dye molecules in the chelate molecule).

The results are listed in Table 10. The results show that, such complexes are fairly stable as indicated by the high values of log K and the free energy changes which are the conditions required for a good metal indicator, even for the 1:2 complexes, which permits the use of these indicators in chelatometric titration methods. The end point being determined spectrophotometrically. The values obtained, Table 10, show that 1:2 species nearly twice as stable as the 1:1 complex.

**Table 10:** Experimental data of the apparent stability constants of Co (II), Ni (II), Cu (II), Zn (II) chelates with PAP azo-dyes (Ia and lb)

Metal	Method	Ia				Ib			
		n = 1		n = 2		n = 1		n = 2	
		Log ${\beta }_{1}$	− ΔG $^\circ$ k.Cal.mol^−1^	Log ${\beta }_{1}$	− ΔG $^\circ$ k.Cal.mol^−1^	Log ${\beta }_{1}$	− ΔG $^\circ$ k.Cal.mol^−1^	Log ${\beta }_{1}$	− ΔG $^\circ$ k.Cal.mol^−1^
Co(II)	MR	5.20	7.14	10.20	14.00	5.08	6.97	9.30	12.76
	CV	5.07	6.96	10.70	14.60	–	–	9.40	12.90
Ni(II)	MR	5.03	6.96	10.30	14.14	5.10	7.00	9.50	13.40
	CV	5.20	7.14	10.48	14.38	–	–	9.90	13.59
Cu(II)	MR	5.80	7.96	10.70	14.69	–	–	10.60	14.55
	CV	–	–	10.05	13.79	5.19	7.12	10.40	14.27
Zn(II)	MR	5.10	7.00	10.50	14.41	5.10	7	9.90	13.59
	CV	5.20	7.14	10.06	13.81	5.16	7.08	9.50	13.04

*n* the stoichiometry of chelate, *β* the stability constant

Standard uncertainties, u, are: u(Log ${\beta }_{1}$) = 0.05, u(ΔG $^\circ$) = 0.04

## Data Availability

All data generated or analyzed during this study are included in this published article.
